# Systems genetics of the *Drosophila* metabolome

**DOI:** 10.1101/gr.243030.118

**Published:** 2020-03

**Authors:** Shanshan Zhou, Fabio Morgante, Matthew S. Geisz, Junwu Ma, Robert R.H. Anholt, Trudy F.C. Mackay

**Affiliations:** 1 Program in Genetics, W.M. Keck Center for Behavioral Biology and Department of Biological Sciences, North Carolina State University, Raleigh, North Carolina 27695, USA;; 2 Key Laboratory for Animal Biotechnology of Jiangxi Province and the Ministry of Agriculture of China, JiangXi Agricultural University, JiangXi, China

## Abstract

How effects of DNA sequence variants are transmitted through intermediate endophenotypes to modulate organismal traits remains a central question in quantitative genetics. This problem can be addressed through a systems approach in a population in which genetic polymorphisms, gene expression traits, metabolites, and complex phenotypes can be evaluated on the same genotypes. Here, we focused on the metabolome, which represents the most proximal link between genetic variation and organismal phenotype, and quantified metabolite levels in 40 lines of the *Drosophila melanogaster* Genetic Reference Panel. We identified sex-specific modules of genetically correlated metabolites and constructed networks that integrate DNA sequence variation and variation in gene expression with variation in metabolites and organismal traits, including starvation stress resistance and male aggression. Finally, we asked to what extent SNPs and metabolites can predict trait phenotypes and generated trait- and sex-specific prediction models that provide novel insights about the metabolomic underpinnings of complex phenotypes.

Defining the genotype-phenotype relationship for complex traits is of central importance for agriculture, precision medicine, and exploring the mechanisms that drive adaptive evolution. However, understanding how genetic variation for complex traits in heterogeneous populations correlates with phenotypic variation remains challenging, due to *trans* regulation, pleiotropy, epistasis, genome-by-environment interactions, epigenetic modifications, and the nonlinear relationships between transcript abundances and corresponding protein levels (Mackay and Anholt 2006, 2007; Manolio et al. 2009; Anholt and Mackay 2018). How effects of DNA sequence variants are transmitted through intermediate endophenotypes to modulate organismal traits is a central question. Here, we address this issue by focusing on the relationship between genomic variation, gene expression, and the metabolome.

The metabolome represents the most proximal link between genetic variation and organismal phenotype. Metabolites are the building blocks for DNA, RNA, proteins, complex lipids, and carbohydrates, serve as cofactors for enzymes, and mediate energy production and signaling processes. The composition and dynamics of the metabolome represent the integrated output of genetic, transcriptomic, and proteomic variation.

Advancing our understanding of genotype-phenotype relationships of complex traits requires systems genetic analyses that incorporate genetic variation with variation in gene expression traits, the metabolome, and complex trait phenotypes in a population with replicated genotypes. Such comprehensive studies are challenging in human populations but can be performed in model organisms that allow precise control of the genetic background and environmental rearing conditions (Joyce and Palsson 2006; Lehner 2013; Civelek and Lusis 2014). The *Drosophila melanogaster* Genetic Reference Panel (DGRP), a wild-derived population of fully sequenced inbred lines, enables comprehensive systems genetic analyses of complex traits to be performed on replicated genotypes (Mackay et al. 2012; Huang et al. 2014; Mackay and Huang 2018). In addition, unlike studies that rely on linkage mapping, rapid decay of linkage disequilibrium within the DGRP (Huang et al. 2014) enables precise mapping.

Here, we used 40 DGRP lines, sexes separately, to identify genetically variable metabolites and metabolomic modules associated with variation of organismal phenotypes. We constructed networks that integrate DNA sequence variation and variation in gene expression with variation in metabolites and organismal traits. Finally, we explored phenotypic prediction models based on variable metabolites.

## Results

### Phenotypic variation of the metabolome

**Figure 1.**
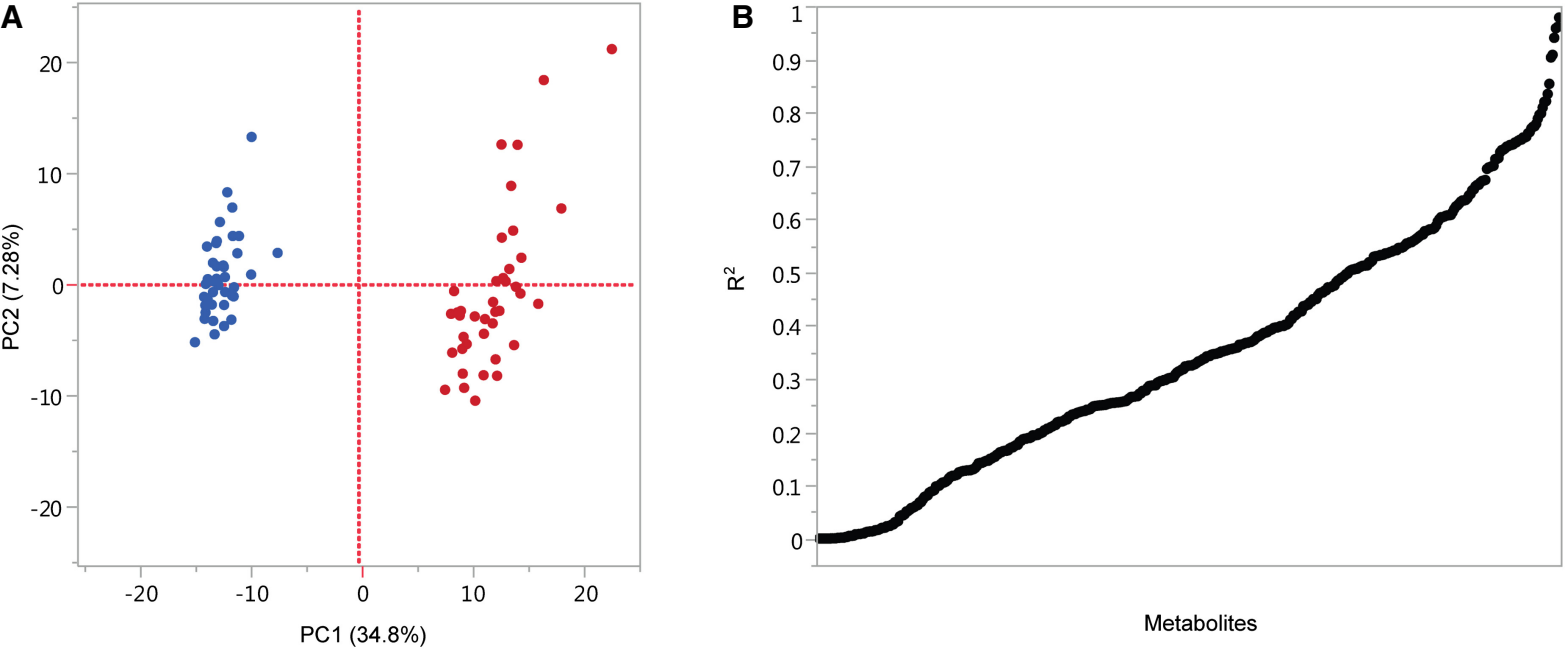
Principal component analysis (PCA) of variation in the metabolome across 40 DGRP lines (*A*) and correlations across the lines between females and males for each metabolite (*B*).

We used ultraperformance liquid chromatography–tandem mass spectrometry to quantify variation in the metabolome of 3- to 7-d-old flies across 40 DGRP lines. We identified 453 metabolites which represent eight “super pathways” including metabolic pathways for lipids, xenobiotics, nucleotides, amino acids, energy metabolism, carbohydrates, cofactors and vitamins, and peptides (Supplemental Table S1). Among these, 53 metabolites were confidently detected without formally documented standards (Supplemental Table S1). We performed principal component analysis (PCA) and observed strong sexual dimorphism of metabolite abundances between females and males associated with the first principal component, explaining 34.8% of the total variation (Fig. 1A). Each of the remaining principal components explains <8% of the total variation. In addition, we found extensive variation in correlations among individual metabolites between females and males across the lines (Fig. 1B). Squared coefficients range from 0 to 0.91. Four metabolites, 1-(1-enyl-palmitoyl)-2-linoleoyl-GPC (P-16:0/18:2), 1-(1-enyl-palmitoyl)-2-palmitoleoyl-GPC (P-16:0/16:1), glycerol 2-phosphate, and mevalonate 5-phosphate, were identified only in males and 1-stearoyl-GPI (18:0) was identified only in females.

### Genetic variation of the metabolome

Mixed-effect ANOVAs quantifying the effects of DGRP line, sex, and the line by sex interaction effects identified 380 metabolites that were significantly variable across lines (FDR < 0.05), 381 metabolites with different abundances between females and males, and 172 metabolites with a significant line by sex interaction (Supplemental Table S2). Among these, 118 metabolites are significant for all three terms. The average broad sense heritability (*H*^2^) of all metabolites is *H*^2^ = 0.43, which indicates a considerable genetic contribution to the observed phenotypic variation. Since there were extensive differences between males and females for most of the metabolites, we also performed reduced model ANOVAs for sexes separately. These analyses identified 371 metabolites in females and 355 metabolites in males that are variable (FDR < 0.05) across different genetic backgrounds (Supplemental Table S3). We focused on these metabolites for downstream analyses of males and females, separately.

In addition, 82 metabolites in females and 98 metabolites in males were not genetically variable, and 43 of these metabolites were common in both sexes. These metabolites are likely tightly regulated at steady state. In females, they include common precursors for fatty acid biosynthesis (malonate and methylmalonate), building blocks for nucleic acids (inosine, guanosine, cytosine, and uridine), intermediates of the tricarboxylic acid cycle (malate and oxaloacetate), glycine, the cofactor nicotinamide, 3 hydroxybutyrate, and a range of complex phospholipids. Glycine, nicotinamide, malate, oxaloacetate, inosine, and guanosine also were not genetically variable in males, in addition to the coenzyme A precursor phosphopantetheine, the vitamin B6 precursor pyridoxal, carnitine, glutamate, and various complex phospholipids.

### Modular organization of the genetically variable metabolome

To search for interacting sets of metabolites based on correlation structure, we performed modulated modularity clustering (Stone and Ayroles 2009) for females and males separately using all variable metabolites.

**Figure 2.**
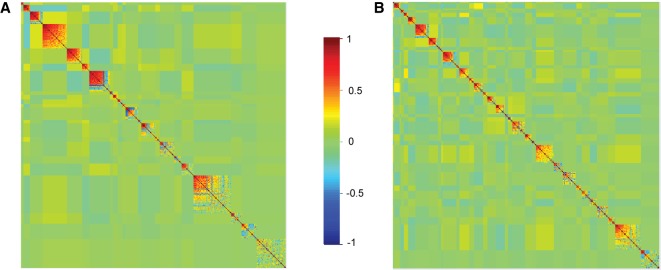
Modulated modularity clustering of metabolites across the 40 DGRP lines in females (*A*) and males (*B*). Modules are ordered from the *top left* corner to the *bottom right* corner based on the average absolute correlation of each module.

We identified 22 modules with correlated metabolites in females (Fig. 2A). Most of the modules contain metabolites predominantly from one or two super pathways, reflecting functional connectivity (Supplemental Table S4A), including lipid metabolism, carbohydrate, peptide, amino acid, and nucleotide metabolism. Modules 8, 10, 12, 16, and 21 comprise metabolites associated with diverse metabolic processes.

For males, we identified 33 tightly correlated modules (Fig. 2B). Unlike females, about half of the male modules contain metabolites associated with diverse super pathways (Supplemental Table S4B). We did not observe significant correlations between modules for either females or males.

Thus, we found extensive differences between metabolomic profiles as well as individual metabolite abundances between females and males, with females having, on average, larger modules than males.

### Metabolite quantitative trait locus (mQTL) mapping

**Figure 3.**
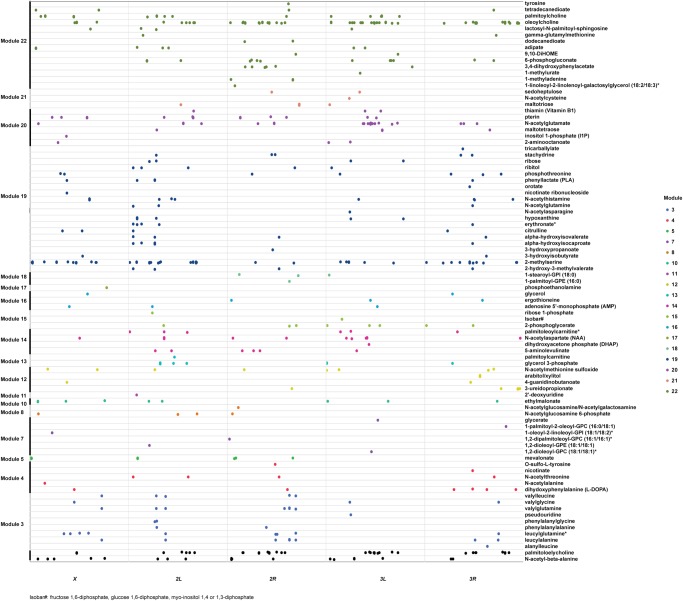
Polymorphic markers associated with variation in metabolites at a Bonferroni-corrected threshold of significance in females. Metabolites and polymorphic markers are ordered according to the modules identified in Figure 2 and color-coded. Black symbols represent polymorphic markers that are associated with metabolites that are not contained in modules.

**Figure 4.**
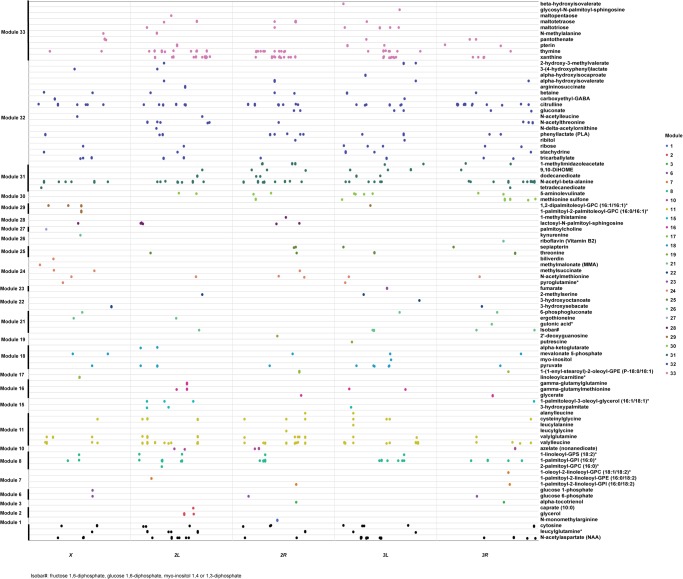
Polymorphic markers associated with variation in metabolites at a Bonferroni-corrected threshold of significance in males. Metabolites and polymorphic markers are ordered according to the modules identified in Figure 2 and color-coded. Black symbols represent polymorphic markers that are associated with metabolites that are not contained in modules.

We identified DNA sequence variants associated with variation in abundance of each metabolite (mQTLs). We tested 1,561,516 bi-allelic single nucleotide polymorphisms and deletions and insertions with the minor allele present in at least four DGRP lines (minor allele frequency [MAF] ≥ 0.1). In females, we identified 754 mQTLs in or near 576 genes and 167 mQTLs in 126 intergenic regions (polymorphisms within 2 kb are considered in the same intergenic region) that were associated with 92 metabolites at a Bonferroni-corrected threshold of *P* ≤ 3.2 × 10^−8^ (Fig. 3; Supplemental Table S5A). In males, we mapped 993 mQTLs in or near 664 genes and 229 mQTLs in 158 intergenic regions associated with 100 metabolites (Fig. 4; Supplemental Table S5B). In females and males, respectively, 808 mQTLs (87.7%) and 1115 mQTLs (91.2%) are associated with only one metabolite. Furthermore, pleiotropic mQTLs are primarily associated with structurally related metabolites, indicating that polymorphisms exert specific effects on variation of individual metabolites. By contrast, each metabolite is associated with an average of 12 and 14 mQTLs in females or males with a median of 5 mQTLs for both sexes. A total of 110 polymorphisms associated with 15 metabolites are common for both sexes.

Most mQTLs are intronic, followed numerically by intergenic, upstream, and downstream mQTLs. There are 77 nonsynonymous coding polymorphisms associated with 39 metabolites. These are in coding regions of 60 genes, 22 of which do not have annotated functions. They are not enriched for specific pathways or functional groups.

Since metabolites are not independent of each other (Fig. 2), we performed PCA on each module of correlated metabolites, separately for males and females. For each module, we retained PCs that explained more than 4% of the variation and added PCs, if needed, to cumulatively explain more than 90% of the variation. We then performed mQTL mapping on each PC from each module. In females, we identified 35 mQTLs in or near 23 genes and five mQTLs in five intergenic regions associated with PCs of seven modules at a Bonferroni-corrected significance threshold (Supplemental Table S6A). In males, we found 27 mQTLs in or near 23 genes and three mQTLs in three intergenic regions associated with PCs of seven modules (Supplemental Table S6B).

To identify mQTLs that are associated both with individual metabolites and module PCs, we first considered the 2033 polymorphisms associated with variation in abundances of individual metabolites at a Bonferroni-corrected threshold. We then relaxed the *P*-value for association of polymorphisms with module PCs to *P* < 2.17 × 10^−6^ to capture the same number of mQTLs: 1021 for female module PCs and 1018 for module PCs, with six of the mQTLs associated with both female and male module PCs (Supplemental Table S6).

**Figure 5.**
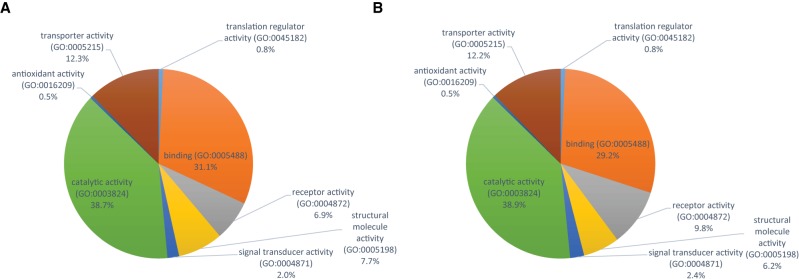
Relative representations of Gene Ontology categories for molecular activities of annotated candidate genes associated with variation in metabolites and module PCs for females (*A*) and males (*B*).

In females, we found only nine mQTLs (0.5%) and 85 genes (7.7%) associated with both individual metabolites and module PCs, while in males there are 32 mQTLs (1.4%) and 172 genes (10.7%) in common to individual metabolites and metabolite PCs. There is little overlap between polymorphisms and genes that are associated with variation in individual metabolites and module PCs. However, in each case their biological functions are enriched in Gene Ontology (GO) categories of neuron differentiation and tissue morphogenesis (Supplemental Table S7), including genes associated with signal transduction, membrane transporters, receptors, and metabolic enzymes (Fig. 5).

### Metabolite-wide association studies (MWAS)

We performed metabolite-wide association studies (MWAS) using Spearman's correlation tests to identify metabolites and metabolomic modules associated with variation of morphological, physiological, and fitness-related phenotypes, including body weight, thorax length, thorax width, starvation resistance, startle response, waking activity, and lifespan for both sexes, as well as inter-male aggression (Jumbo-Lucioni et al. 2010; Huang et al. 2012; Harbison et al. 2013; Ivanov et al. 2015; Shorter et al. 2015). We also assessed free glucose and free glycerol levels along with glycogen, triglyceride, and total protein levels using colorimetric and fluorometric methods (Supplemental Table S8). We observed high correlations between variations in the concentration of glucose and glycerol measured by mass spectrometry, with free glucose and free glycerol measured biochemically in both females and males.

**Figure 6.**
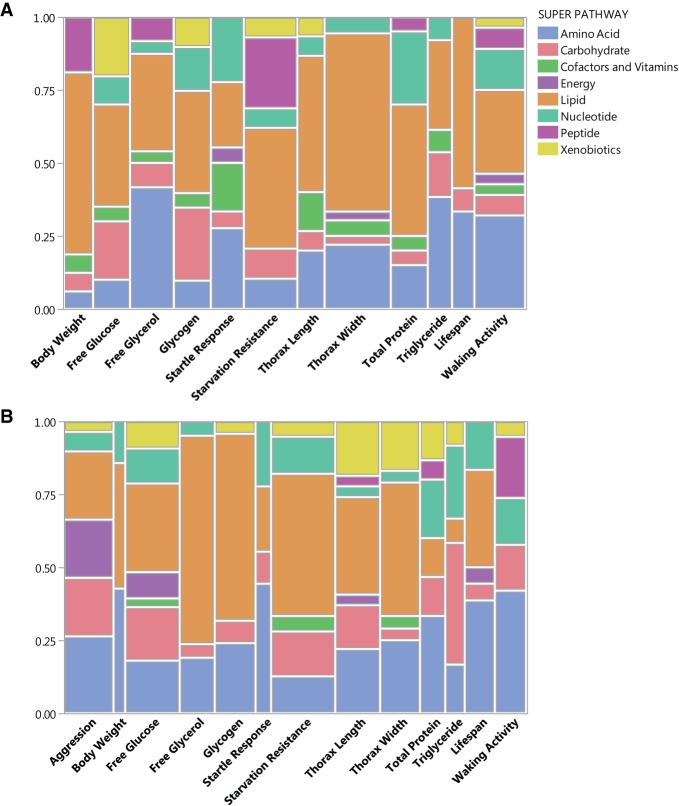
Distribution of metabolic super pathways with metabolites correlated with variation in organismal phenotypes in females (*A*) and males (*B*). The widths of the columns indicate the relative numbers of metabolites correlated with variation of the traits.

In females, we found 157 metabolites that showed significant correlations with the 12 traits, ranging from 12 metabolites that were correlated with lifespan to 36 metabolites that were correlated with thorax width (Supplemental Table S9A). A total of 94 metabolites were uniquely associated with one trait and 63 metabolites were associated with two to four traits. Correlations for most traits involved metabolites across at least five super pathways (Fig. 6A). However, variation in lifespan only correlated with metabolic pathways of lipids, carbohydrates, and amino acids. Variation in body weight and thorax width also correlated predominantly with variation in levels of lipid metabolites (Fig. 6A).

In males, we identified 190 metabolites that are correlated with 13 organismal traits, including aggression (Supplemental Table S9B). Correlated metabolites ranged from seven with body weight to 39 with starvation resistance. We observed that 122 metabolites were correlated with only a single trait, while 65 correlated with two or three traits. As in females, correlations for most traits involved metabolites across at least five super pathways (Fig. 6B). Variation in free glycerol and glycogen correlated predominantly with lipid metabolites, whereas variation of body weight correlated with variation in metabolic pathways of lipids, amino acids, and nucleotides (Fig. 6B).

In addition to individual metabolites, we identified 77 PCs from 21 modules and 92 PCs from 29 modules that were correlated with the same set of organismal traits in females and males, respectively (Supplemental Table S9C, D). Among those, 49 and 59 PCs were uniquely correlated with one trait and the others with two or three traits. The absolute correlation coefficients ranged from |*r*| = 0.31 to |*r*| = 0.60. In both females and males, PCs that correlated with variation in lifespan had the highest average absolute correlation coefficients.

We examined phenotypic correlations between the 13 tested traits and found for both females and males (Supplemental Table S10) that free glycerol was correlated with triglyceride, total protein was correlated with body weight, and thorax width was correlated with thorax length, as would be expected. In males, body weight and thorax length are correlated with free glucose and total protein, whereas in females, body weight is correlated with glycerol and triglyceride levels. Starvation resistance in males is correlated with glycogen and waking activity but negatively correlated with thorax length, whereas in females, starvation resistance is positively correlated with free glucose and lifespan.

**Figure 7.**
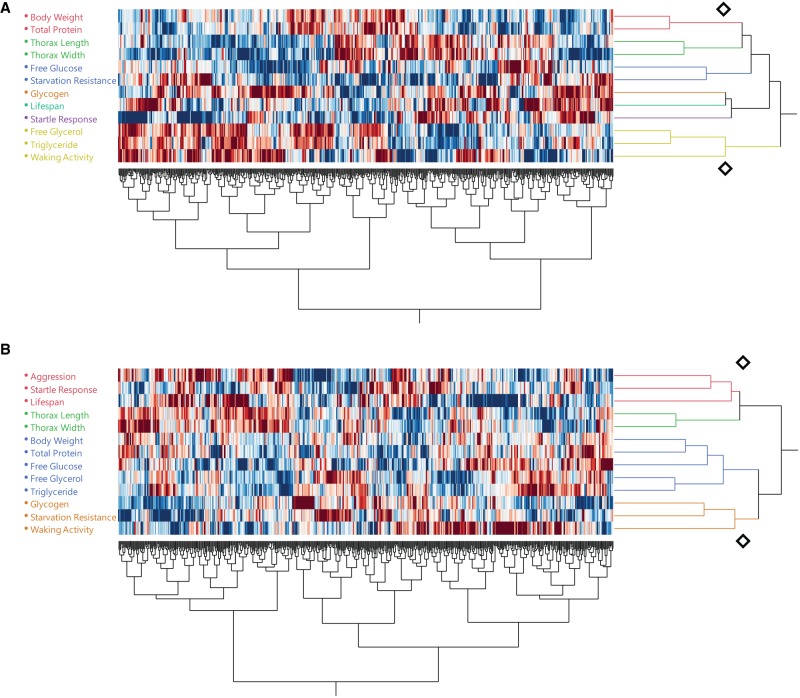
Hierarchical clustering analysis of different traits based on common correlated metabolites and module PCs for females (*A*) and males (*B*). The diamond symbols indicate distances chosen to determine the appropriate number of clusters.

Next, we performed clustering analyses among these traits based on their correlation patterns across metabolites and module PCs to assess to what extent variation in different organismal traits is influenced by common aspects of the organization of the metabolome (Fig. 7). Clustering analyses recapitulate the relationship between correlated traits. These analyses also revealed hidden pleiotropic relationships. For example, aggression is clustered with startle response, and they both are clustered with lifespan in males; waking activity is clustered with triglyceride and free glycerol in females. This clustering is not due to uniformly positive or negative correlations with the same metabolites; rather, it reveals both agonistic and antagonistic pleiotropic relationships which would not be detectable when considering only phenotypic correlations without accounting for their metabolomic associations.

### Networks that incorporate genetic variation in gene expression with variation in metabolites and organismal traits

Transcriptional profiles were also obtained through directional RNA-seq from 39 of the 40 lines (Everett et al. 2020). In total, 17,295 annotated genes and 22,726 novel transcripts were captured. In females, expression of 9640 genes and 1644 novel transcripts were significantly variable across the 39 lines, and 9532 genes and 3204 novel transcripts were differentially expressed in males (Supplemental Table S11).

To exclude correlations caused by extreme lines, we performed Spearman's rank correlations between genetically variable transcripts and metabolites. We began this analysis with the metabolites correlated with each of the different traits, separately for males and females (Supplemental Table S9). We then focused on transcript and metabolite pairs with Spearman's correlation coefficients greater than 0.45 and identified genetic variants associated with variation of both gene expression (eQTLs) and metabolite abundance (mQTLs):meQTLs. We then identified meQTLs that were also associated with each of the organismal quantitative traits (Supplemental Table S12). This enabled us to construct integrated networks (Supplemental Fig. S1).

**Figure 8.**
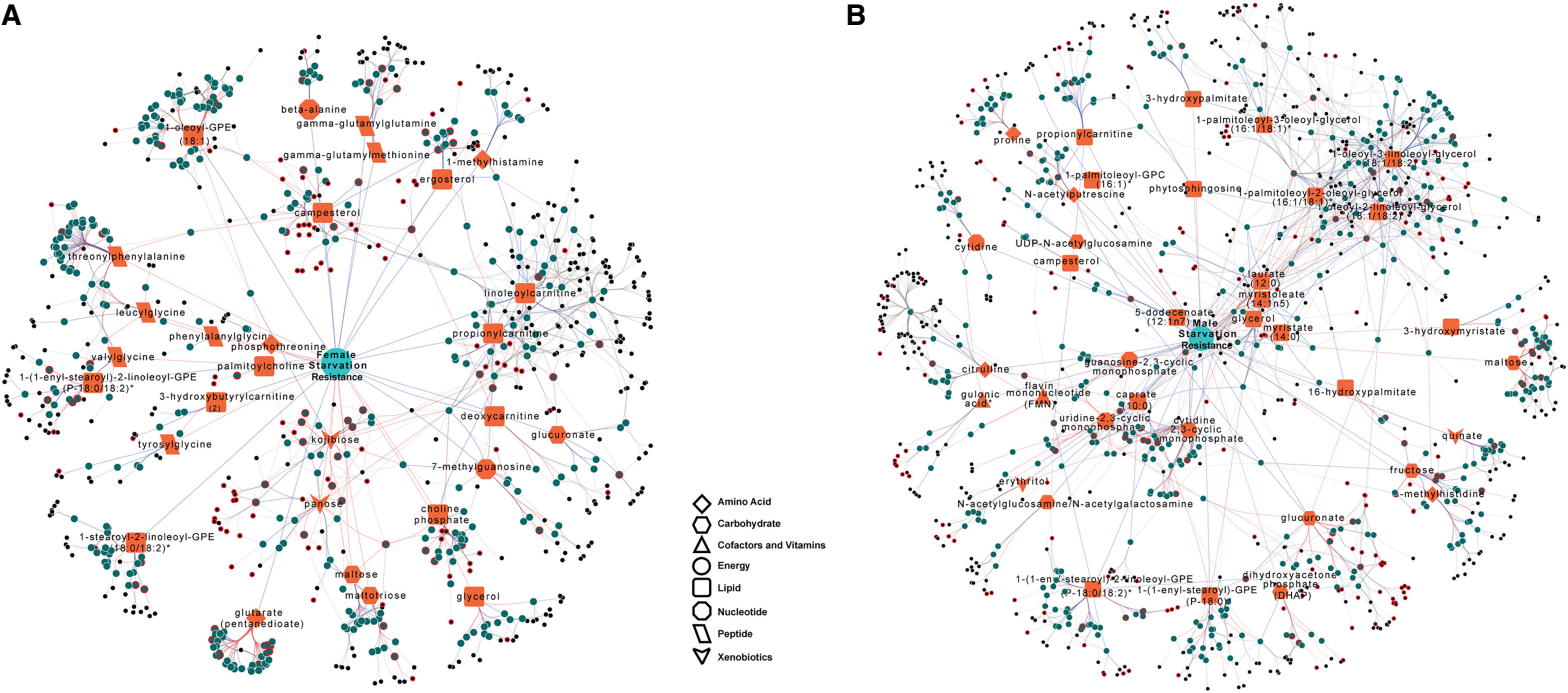
Integrated networks that incorporate polymorphic markers, variation in candidate gene expression, and variation in metabolite abundances associated with variation in starvation resistance for females (*A*) and males (*B*). Orange nodes indicate metabolites correlated with starvation resistance and teal nodes indicate candidate genes correlated with these metabolites. Black nodes indicate mQTL associated with candidate genes. Nodes with red borders indicate a direct association with the organismal phenotype. The different shapes of the orange nodes indicate different metabolic super pathways. Red edges indicate positive correlations, while blue edges represent negative correlations. Black edges connect polymorphic markers with their associated genes. The polymorphic markers, candidate genes, and metabolites presented in the figure are listed in Supplemental Table S12.

**Figure 9.**
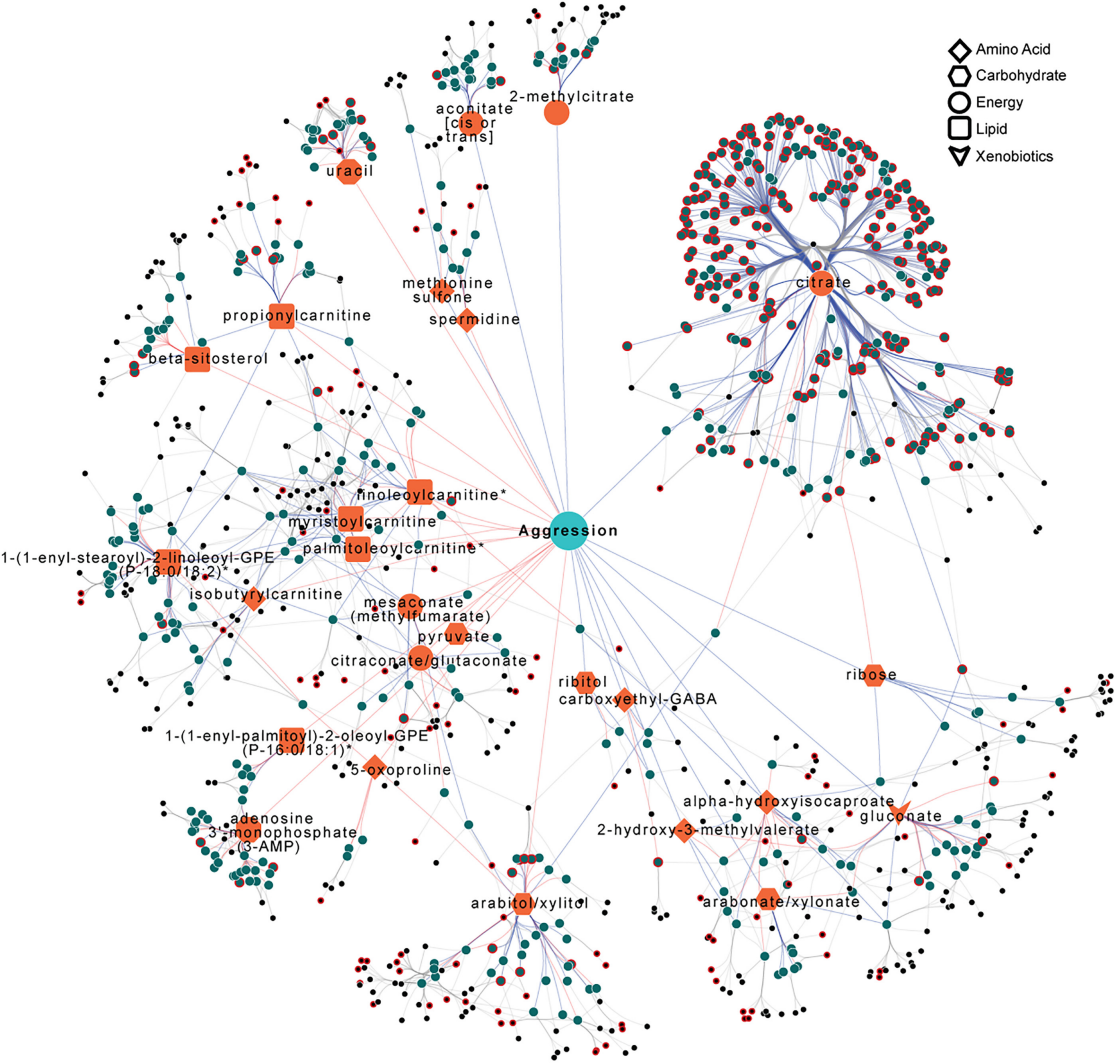
Integrated network that incorporates polymorphic markers, variation in candidate gene expression, and variation in metabolite abundances associated with variation in male aggression. Orange nodes indicate metabolites correlated with aggression and teal nodes indicate candidate genes correlated with these metabolites. Black nodes indicate mQTLs associated with candidate genes. Nodes with red borders indicate a direct association with the organismal phenotype. The different shapes of the orange nodes indicate different metabolic super pathways. Red edges indicate positive correlations, while blue edges represent negative correlations. Black edges connect polymorphic markers with their associated genes. The polymorphic markers, candidate genes, and metabolites presented in the figure are listed in Supplemental Table S12.

We present examples of integrated networks for starvation resistance for females (Fig. 8A) and males (Fig. 8B) and for male aggression (Fig. 9). These networks reveal metabolites that are regulated by multiple gene products, and for starvation resistance, highlight sexual dimorphism (Fig. 8). The integrated network for females shows that metabolites connected by common genetic variants are often members of the same metabolic super pathways (Fig. 8A). In contrast, in males, metabolites connected by common genetic variants often belong to different metabolic super pathways (Fig. 8B). These observations recapitulate the modular organization revealed by modulated modularity clustering (Supplemental Table S4). Pathways featuring peptide and amino acid metabolism are prominent in the female network, whereas lipid metabolism is especially apparent in the male network. Thus, distinctly different genetic and metabolic underpinnings govern variation in starvation resistance in males and females (Fig. 8).

The integrative network for male aggression shows ensembles of metabolites with distinct positive and negative correlations with phenotypic variation. Metabolites directly associated with energy release, including Krebs cycle intermediates and carnitine esters that transport fatty acids into the mitochondria for β-oxidation, feature prominently in the network (Fig. 9).

### Metabolome-based prediction of organismal phenotypes

We asked to what extent genetic variation in metabolites and module PCs can predict organismal phenotypes. We first conducted comparisons between whole genome prediction (using all common SNPs), predictions based on polymorphisms associated with variation in metabolites (mQTL), and metabolite prediction, using best linear unbiased prediction (BLUP) with leave-one-out cross-validation.

**Figure 10.**
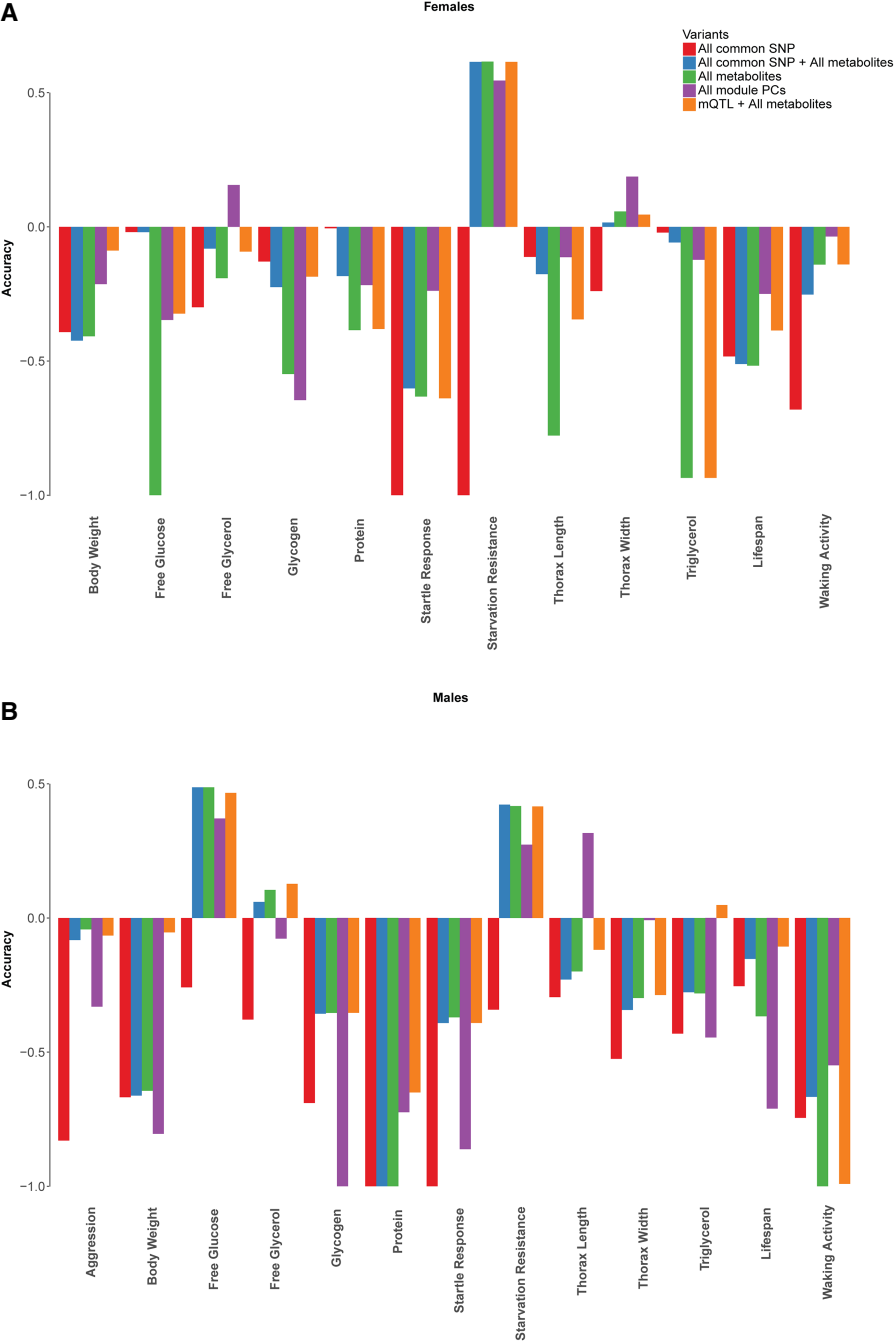
Comparisons of prediction accuracy using genome-wide SNPs with MAF > 0.05, all variable metabolites, common SNPs, and variable metabolites, all module PCs, SNPs associated with variable metabolites or module PCs (mQTLs), and metabolites for females (*A*) and males (*B*).

We compared prediction accuracy using genome-wide SNPs with MAF > 0.05, all variable metabolites, common SNPs and variable metabolites, SNPs associated with variable metabolites (mQTLs), and metabolites. For most of the traits, neither common SNPs nor metabolites provide accurate predictions of the phenotype, except for starvation resistance in both males and females and free glucose levels in males, where analysis of variation in metabolites yielded good predictive values (Fig. 10).

**Figure 11.**
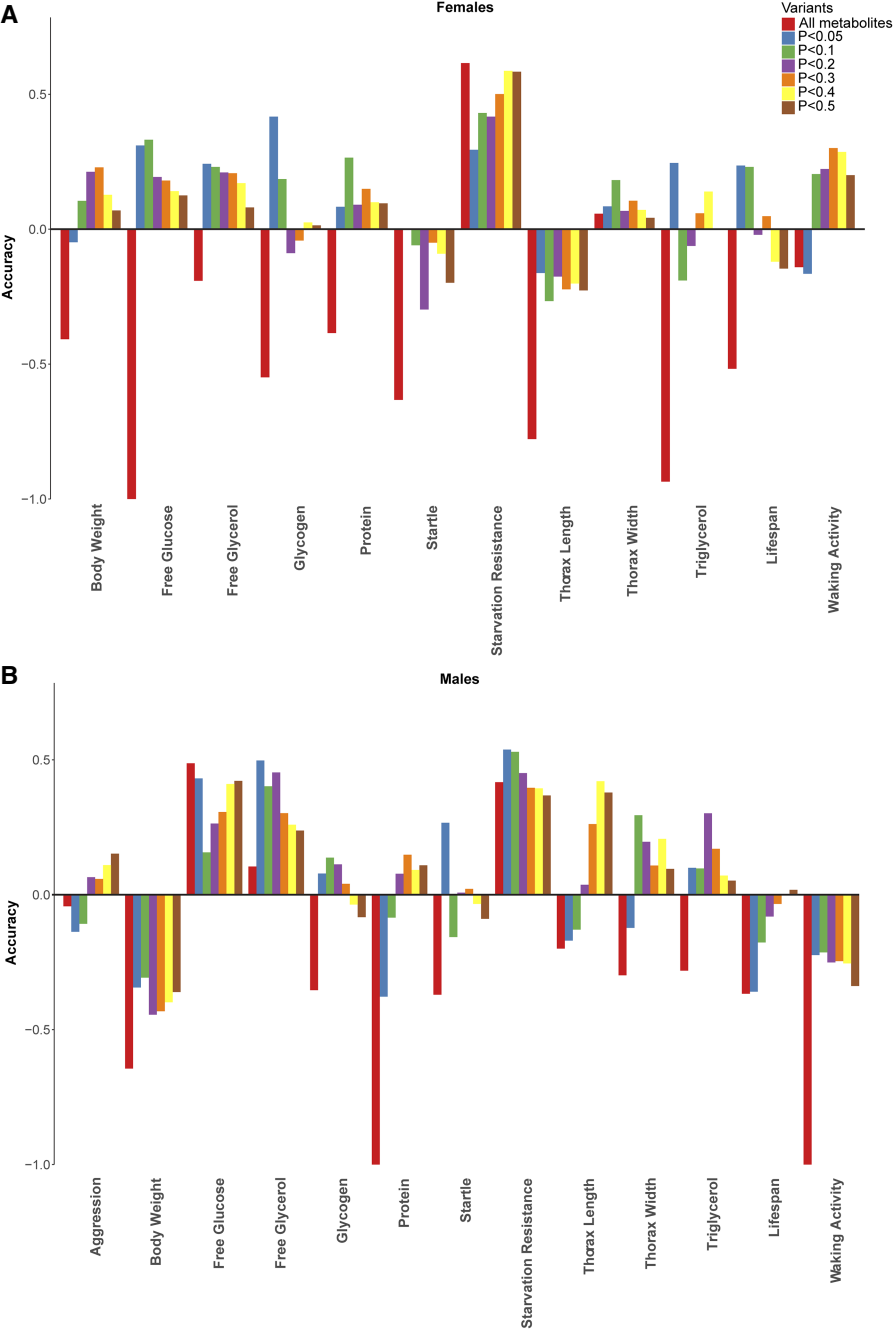
Comparisons of prediction accuracy using all variable metabolites or metabolites enriched for association with particular traits at *P*-values of 0.05, 0.1, 0.2, 0.3, 0.4, and 0.5, for females (*A*) and males (*B*).

Next, we asked whether enriching those metabolites that are associated with variation of a particular phenotype in the model would increase prediction accuracy of that phenotype. We compared all variable metabolites as well as those metabolites enriched for association with particular traits in the training set at *P*-values of 0.05, 0.1, 0.2, 0.3, 0.4, and 0.5. All traits showed improved prediction accuracy using an enriched set of metabolites previously associated with these traits in one or both sexes (Fig. 11). However, the level of enrichment that produces the best prediction accuracy varies for different traits and between sexes.

**Figure 12.**
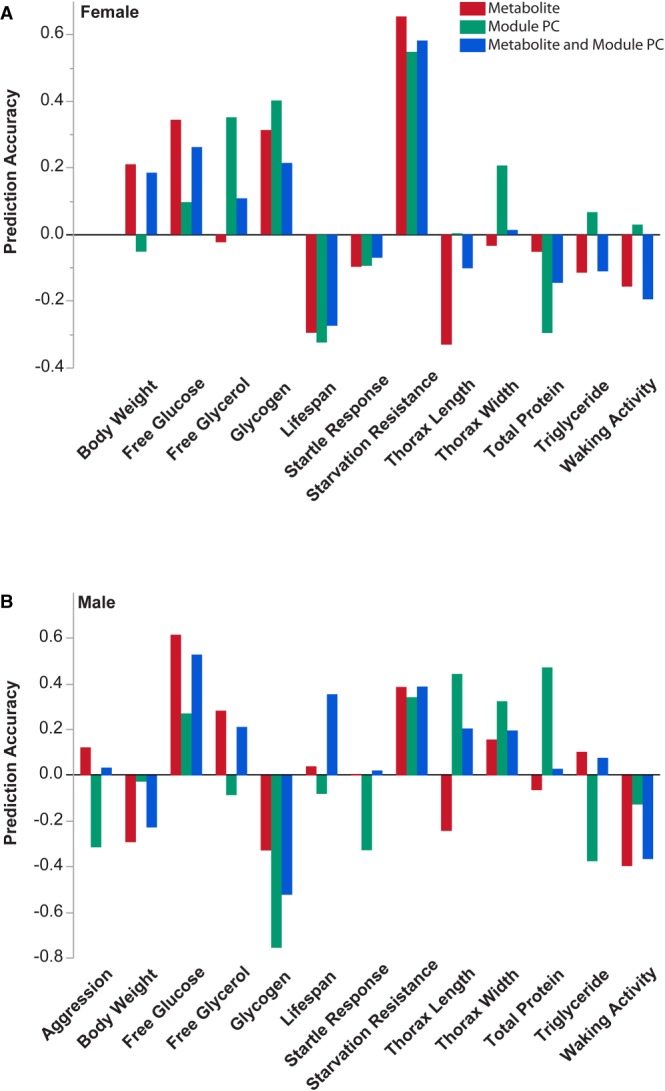
Metabolome-based predictions of organismal traits for females (*A*) and males (*B*). We used the elastic net regularization and leave-one-out cross-validation to enrich metabolites and predict phenotypic values. Prediction accuracy is estimated as the correlation between predicted and actual values. The blue, red, and green bars, respectively, represent models with training sets of metabolites and module PCs combined, or metabolites and PCs separately.

We used the elastic net regularization to build trait-specific models, separately for males and females. In addition, we also used metabolomic module PCs to predict phenotypes and also combined both individual metabolites and module PCs to see whether there would be an improvement in prediction accuracy (Fig. 12). We found that the combination of individual metabolites and module PCs did not increase prediction accuracies over the better of the metabolite and module PC models.

In summary, for most traits enrichment for metabolites known to be associated with variation in a particular phenotype in the training set increases the prediction accuracy for that phenotype. Finally, prediction models are trait-specific and sex-specific, indicating that the metabolomic underpinnings that contribute to phenotypic variation are different for different traits and between the sexes.

## Discussion

Previous studies have associated genetic variation with metabolic phenotypes in human populations (Gieger et al. 2008; Illig et al. 2010; Suhre et al. 2011; Shin et al. 2014) and model organisms (Klenø et al. 2004; Gilliland et al. 2006; Keurentjes et al. 2006; Martin et al. 2007; Wentzell et al. 2007; Schauer et al. 2008; Fu et al. 2009; Riedelsheimer et al. 2012; Breunig et al. 2014; Reed et al. 2014; Williams et al. 2015; Dumas et al. 2016; Fernie and Tohge 2017; Swain-Lenz et al. 2017). Disease-centered high dimensional multi-omic analyses have provided insight into the relationship between genetic variation and susceptibility to diseases (Hood et al. 2004; Prabakaran et al. 2004; Ibrahim and Gold 2005; Samuel et al. 2008). However, to date, a comprehensive integration of genome-wide variants with variation in gene expression, the metabolome, and organismal phenotype along with metabolome-based phenotypic prediction has not been reported for any genetically well-defined model organism population, and few studies have attempted to predict phenotypes based on variation in the metabolome while accounting for interdependence of metabolites. Furthermore, metabolome-centered genetic studies rarely compare differences between females and males. Human studies have been limited by sample sizes (Illig et al. 2010; Shin et al. 2014), plant and yeast models are not amenable to studies of sex differences (Chan et al. 2010; Breunig et al. 2014), and most integrative studies in *Drosophila* have been performed at the larval stage (Reed et al. 2014; Williams et al. 2015). Our study represents the first comprehensive systems genetics analysis that tracks sexual dimorphism at each level of analysis, from genetic associations to the metabolome and organismal phenotypes.

We observed extensive sexual dimorphism in the modular organization of the metabolome, in line with previous studies (Hoffman et al. 2014), as well as in the composition of networks that integrate genomic and metabolomic variation with variation in organismal phenotypes. For example, evidence that energy metabolism is managed differently between the sexes comes from our observation that body weight in males is correlated with glucose and protein levels, whereas in females it is correlated with glycerol and triglyceride levels.

A genome-wide association study of natural variation in the metabolome of *Arabidopsis thaliana* found that genetic variants associated with variation in metabolite levels occur as nonrandomly distributed hotspots in genomic regions that may have undergone selective sweeps (Keurentjes et al. 2006; Wentzell et al. 2007; Lisec et al. 2008; Rowe et al. 2008; Chan et al. 2010). We did not observe evidence for such hotspots in our MWAS. Furthermore, whereas eQTL in *A. thaliana* corresponded poorly with metabolite levels (Fu et al. 2009), we observed substantial concordance between eQTL and variation in metabolite abundances, which is consistent with findings in human studies (Shin et al. 2014).

Most DGRP lines harbor segregating inversions, which are islands of heterozygosity. In addition, ∼50% of the DGRP lines are infected with the endosymbiont *Wolbachia pipientis* (Huang et al. 2014). Inversions and *Wolbachia* infection can affect organismal phenotypes and possibly metabolite variation; further, all segregating sites must be treated as missing data in these analyses. For these reasons, we selected 40 unrelated lines that are free of inversions and *Wolbachia*. Increasing the sample size to include more DGRP lines would provide greater statistical power, which might expand the networks presented in Figures 8 and 9. However, the scope of the present study proved sufficient to resolve the modular organization of the metabolome and its relationship to both genomic variants and variation in complex traits.

We opted to focus our studies on whole flies, since complex traits are manifestations of the entire individual. The organization of the metabolome, however, is likely to vary among different tissues (Chintapalli et al. 2013), and further studies would be needed to provide a detailed documentation of tissue-specific specializations of the metabolome.

The integrative networks we derived (Figs. 8, 9) visualize the complex interconnections between meQTL, eQTL, metabolites, and organismal traits and enable identification of coregulated metabolites and pleiotropic relationships. These networks are biologically plausible. The network that underlies male aggression illustrates the dependence of aggressive behavior on energy supply, highlighting Krebs cycle intermediates and carnitine esters that transport fatty acids into the mitochondria for β-oxidation (Fig. 9). Networks associated with starvation resistance demonstrate how different genomic regulation and metabolic underpinnings govern variation in starvation resistance in males and females (Fig. 8).

It is of interest that neural and tissue development are enriched Gene Ontology categories associated with variation in the metabolome, suggesting that developmentally induced variation plays a role in determining variation in the adult metabolome. Previous studies have shown that cellular metabolism plays a critical role in the differentiation of neural stem cells (Knobloch and Jessberger 2017). While quiescent stem cells mostly rely on glycolysis, proliferating stem cells switch to lipogenesis (Chorna et al. 2013; Knobloch et al. 2013).

Whereas genetically variable metabolites have substantial heritabilities, environmental effects on the total variance cannot be ignored. The studies presented here do not capture the dynamics of the metabolome in response to environmental or physiological changes but provide a snapshot of the relationships between the genome, metabolome, and organismal phenotypes at a single controlled age and rearing environment.

Although we used univariate correlation in our network analyses, we are aware that gene-gene interactions and the interdependence of metabolic pathways give rise to nonlinear relationships; for example, phosphorylation of enzymes by polymorphic genes that encode kinases may precipitate indirect wide-ranging effects on metabolite abundances. In fact, we found that all meQTL identified in our networks are *trans* eQTL to genes correlated with metabolites. This also reflects the complex interactions at the level of the genome, transcriptome, and proteome, which are ultimately channeled to the metabolome, which is most proximal to the organismal phenotype. Thus, the metabolome can be viewed as a mechanistic conduit that translates genetic variation into variation in organismal phenotypes.

Finally, we are aware that our metabolome-based prediction study is based on a small sample size of 40 lines and that larger sample sizes could improve the accuracy of metabolome-based prediction. However, our observations constitute a “proof-of-concept” that metabolites can be good predictors of phenotypes and that even with a small training set, phenotypic prediction based on variation of the metabolome can yield greater accuracy than predictions based on genetic variants alone.

## Methods

### Fly stocks

We used 40 sequenced, wild-derived, inbred DGRP lines (Mackay et al. 2012; Huang et al. 2014): DGRP_41, DGRP_ 42, DGRP_45, DGRP_59, DGRP_83, DGRP_91, DGRP_129, DGRP_158, DGRP_177, DGRP_195, DGRP_208, DGRP_217, DGRP_228, DGRP_229, DGRP_239, DGRP_307, DGRP_315, DGRP_357, DGRP_367, DGRP_371, DGRP_375, DGRP_379, DGRP_385, DGRP_391, DGRP_392, DGRP_399, DGRP_427, DGRP_439, DGRP_491, DGRP_508, DGRP_509, DGRP_517, DGRP_703, DGRP_757, DGRP_765, DGRP_774, DGRP_799, DGRP_808, DGRP_843, DGRP_900. These 40 lines are minimally related, maximally homozygous, have standard karyotypes for all common polymorphic inversions, and are not infected with *Wolbachia pipientis*. Fly lines were reared on cornmeal-molasses-yeast medium at 25°C under a 12-h light-dark cycle. We collected three replicates of 100 flies from each line, sexes separately, which were flash-frozen and stored at −80°C. All 240 samples were sent to Metabolon, Inc. for metabolomic profiling.

### Metabolomic profiling

Samples were prepared by Metabolon, Inc. using the automated MicroLab STAR system from Hamilton Company. Several recovery standards were added prior to the first step in the extraction process for QC purposes. To remove protein, dissociate small molecules bound to protein or trapped in the precipitated protein matrix, and to recover chemically diverse metabolites, proteins were precipitated with methanol under vigorous shaking for 2 min (Glen Mills GenoGrinder 2000), followed by centrifugation. The resulting extract was divided into five fractions: two for analysis by two separate reverse phase (RP)/UPLC-MS/MS methods with positive ion mode electrospray ionization (ESI), one for analysis by RP/UPLC-MS/MS with negative ion mode ESI, one for analysis by HILIC/UPLC-MS/MS with negative ion mode ESI, and one sample was reserved for backup. Samples were placed briefly on a TurboVap (Zymark) to remove the organic solvent. The sample extracts were stored overnight under nitrogen before preparation for analysis.

Raw data were extracted, peak-identified, and QC-processed using Metabolon's hardware and software. Compounds were identified by comparison to library entries of purified standards or recurrent unknown entities. Peaks were quantified using area-under-the-curve. A data normalization step was performed to correct variation resulting from instrument inter-day tuning differences. Each compound was corrected in run-day blocks by registering the medians to equal one (1.00) and normalizing each data point proportionately. The detailed procedure for metabolomic profiling from Metabolon, Inc. is included as Supplemental Methods.

### Statistical and quantitative genetic analysis

We analyzed variation of metabolites among DGRP lines using the ANOVA model *Y = μ + L + S + L×S + ɛ*, where *Y* is the observed value, *μ* the mean*, L* (line) is a random effect, *S* (sex) is fixed, and *ɛ* is the error variance. We also analyzed variation of metabolites for sexes separately, using the reduced model *Y = μ + L + ɛ*. We estimated variance components with the restricted maximum likelihood method and calculated broad sense heritability as $H^{2}=\;\;\sigma_{G}^{2}/\sigma_{P}^{2}$, where $\sigma_{G}^{2}$ is the total genetic variation ($\sigma_{L}^{2}+\;\sigma_{L\times S}^{2}$) and $\sigma_{P}^{2}$ is the total phenotypic variation, where $\sigma_{P}^{2}=\;\sigma_{G}^{2}+\;\sigma_{\varepsilon }^{2}$ (Falconer and Mackay 1996).

To assess correlations between metabolites, we performed modulated modularity clustering on genetically variable metabolites (FDR < 0.05 from reduced ANOVA models) for sexes separately (Stone and Ayroles 2009). We then conducted principal component analyses for each module. We retained PCs that cumulatively explained >90% of the variation for each module for subsequent analyses.

### Genome-wide association

To obtain metabolite QTL (mQTL), we performed GWA analyses for individual metabolites, sexes separately. We used 1,561,516 bi-allelic single nucleotide polymorphisms and deletions and insertions with minor allele frequencies greater than 0.1, using the DGRP pipeline (Huang et al. 2014). We also performed GWA analyses for each module-PC to account for interacting metabolites.

### Quantitative trait phenotypes

We retrieved phenotypic data documented from previous publications on the same fly lines for starvation resistance, startle response, waking activity, and virgin lifespan for both sexes, as well as inter-male aggression (Harbison et al. 2004, 2013; Jumbo-Lucioni et al. 2010; Huang et al. 2012; Ivanov et al. 2015; Shorter et al. 2015).

To measure body weight and size, we collected 10 replicates of 10 flies per line and sex into preweighed 1.7-ml tubes and weighed and flash-froze them for downstream analyses. Virgin flies were used to avoid body weight variation due to variation in egg production. In addition, we measured thorax length and thorax width as metrics for body size.

Frozen flies were homogenized in 250 μL Dulbecco's phosphate-buffered saline, and after gentle centrifugation, supernatants were collected for measurements of free glucose, glycogen, free glycerol, triglyceride, and total protein (further diluted 10-fold). For free glucose and glycogen, samples were denatured at 95°C for 25 min to prevent glycogenolysis. Measurements were done following protocols provided by the Glycogen Colorimetric/Fluorometric Assay Kit (BioVision). For free glycerol and triglyceride, we used the Serum Triglyceride Determination Kit (Sigma Aldrich), and incubated samples with the Triglyceride Reagent for 1 h at 37°C. For total protein measurement, we used the Qubit Protein Assay Kit (Thermo Fisher Scientific).

### Correlations between genetic variants, metabolites, and organismal phenotypes

We identified metabolites correlated with different phenotypes using Spearman's correlations at a nominal *P*-value < 0.05. We identified genes correlated with these metabolites at a Spearman's correlation coefficient threshold |*r*| > 0.45. Next, we identified mQTLs that were also associated with these genes for each metabolite at a metabolite-specific Bonferroni threshold [*P* < 0.05/(number of mQTLs associated with the particular metabolite)]. For each trait, genetic polymorphisms, transcripts, and metabolites generated from the above analyses were used to construct integrated networks. Polymorphisms and genes were highlighted if they were directly associated or correlated with the focal trait at a nominal *P*-value < 0.05.

### Metabolome-based prediction

#### Standard BLUP analysis

The best linear unbiased predictor was used to predict phenotypes (Robinson 1991). It is a linear mixed model where the covariance among the random effects is modeled through the use of one or more kernel matrices. In the present studies, several kernels that measure the similarity among lines based on different features were used. The features consisted of: all common SNPs, all metabolites, all module PCs and mQTLs (associated with single metabolites or module PCs).

Kernels for each feature type were built as **K** = **WW**′/*p* where **W** is a centered and scaled *n* × *p* feature matrix, *n* is the number of lines, and *p* is the number of features (Guo et al. 2016). One or two kernel BLUP models were implemented as follows:

1. One kernel model: **y** = 1*μ* + **g**_**K**_ + **e**, where **y** is an *n*-vector of line mean phenotype, **1** is an *n*-vector of ones, *µ* is the population mean, **g_K_** is an *n*-vector of random line effects $\left[g_{K}∼N(0,K\sigma_{K}^{2})\right]$, and **e** is an *n*-vector of random residual effects$\left[e∼N(0,I\sigma_{e}^{2})\right]$. **K** is a kernel from the list above; **I** is the identity matrix.
2. Two kernel model: **y** = 1*μ* + **g**_**K**1_ + **g**_**K**2_ + **e**, where **y** is an *n*-vector of line mean phenotypes, **1** is an *n*-vector of ones, *µ* is the population mean, **g_K1_** is an *n*-vector of random line effects associated with $K_{1}[g_{K1}∼N(0,K_{1}\sigma_{K1}^{2})]$, **g_K2_** is an *n*-vector of random line effects associated with $K_{2}[g_{K2}∼N(0,K_{2}\sigma_{K2}^{2})]$, and **e** is an *n*-vector of random residual effects $\left[e∼N(0,I\sigma_{e}^{2})\right]$. **K**_1_ and **K**_2_ are two kernels from the list above; **I** is the identity matrix.

In order to avoid overfitting and to maximize the power to estimate variance components given the small sample size, all the models were implemented in a leave-one-out cross-validation setting. At each round of the cross-validation, one line was removed from the training set where the variance components were estimated. Using the estimated variance components, the phenotype for the omitted line, that is, test set, was predicted. Accuracy of prediction was evaluated as the correlation coefficient between true and predicted phenotypes.

#### Combined MWAS-BLUP analysis

To parse out the true signal from noise in a trait-specific manner, a combined mapping and prediction analysis was performed with single metabolites. At each round of cross-validation, a single metabolite regression (MWAS) was performed in the training set using a linear model. The metabolites with *P* < *x* (with *x* = 0.5; 0.4; 0.3; 0.2; 0.1; 0.05) were selected and used to build a kernel as described in the previous section. Variance components were still estimated in the training set, and the phenotype of the line in the test set was predicted using the standard BLUP procedure. Accuracy of prediction was again evaluated as the correlation coefficient between true and predicted phenotypes.

#### Elastic net analysis

To identify the maximum prediction accuracy from metabolites and module PCs, we also performed predictions using elastic net regularization (Zou and Hastie 2005). We used individual metabolites, module PCs, and individual metabolite and module PC data combined for phenotype prediction and identified λ and α values through grid-searches that produced the highest prediction accuracies.

## Data access

DGRP lines are available from the *Drosophila* stock center (Bloomington, IN). All raw and processed sequencing data generated in this study have been submitted to the NCBI Gene Expression Omnibus (GEO; https://www.ncbi.nlm.nih.gov/geo/) under accession number GSE117850.

## Competing interest statement

The authors declare no competing interests.

## Supplementary Material

Supplemental Material
