# Supplementary material for: Systems genetics of the Drosophila metabolome
Source: Genome Res. 2020 Mar;30(3):392–405. doi: 10.1101/gr.243030.118 (PMC7111526; doi:10.1101/gr.243030.118)
Supplement: Supplemental Material [file supp_30_3_392__index.html]

Systems genetics of the Drosophila metabolome — Supplemental Material 

# Systems genetics of the *Drosophila* metabolome

## Supplemental Material

- Supplemental\_Fig\_S1.pdf.pdf
- Supplemental\_Methods.docx.docx
- Supplemental\_Table\_S1.xlsx.xlsx
- Supplemental\_Table\_S2.xlsx.xlsx
- Supplemental\_Table\_S3.xlsx.xlsx
- Supplemental\_Table\_S4.xlsx.xlsx
- Supplemental\_Table\_S5.xlsx.xlsx
- Supplemental\_Table\_S6.xlsx.xlsx
- Supplemental\_Table\_S7.xlsx.xlsx
- Supplemental\_Table\_S8.xlsx.xlsx
- Supplemental\_Table\_S9.xlsx.xlsx
- Supplemental\_Table\_S10.xlsx.xlsx
- Supplemental\_Table\_S11.xlsx.xlsx
- Supplemental\_Table\_S12.xlsx.xlsx
