# Supplementary material for: Systems genetics of the Drosophila metabolome
Source: Genome Res. 2020 Mar;30(3):392–405. doi: 10.1101/gr.243030.118 (PMC7111526; doi:10.1101/gr.243030.118)
Supplement: Supplemental Material [file supp_gr.243030.118_Supplemental_Fig_S1.pdf.pdf]

Supplemental Figure S1: Integrated networks that incorporate polymorphic markers, variation in candidate gene expression and variation in metabolite abundances associated with variation in organismal phenotypes. Orange nodes indicate metabolites correlated with the organismal phenotype and teal nodes indicate candidate genes correlated with these metabolites. Black nodes indicate mQTL associated with candidate genes. Nodes with red borders indicate a direct association with the organismal phenotype. The different shapes of the orange nodes indicate different metabolic super pathways. Red edges indicate positive correlations, while blue edges represent negative correlations. Black edges connect polymorphic markers with their associated genes. The polymorphic markers, candidate genes and metabolites presented in the figure are listed in Supplementary File S12.

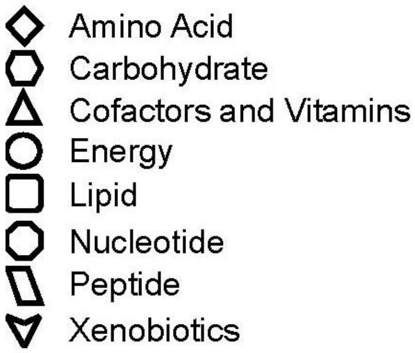

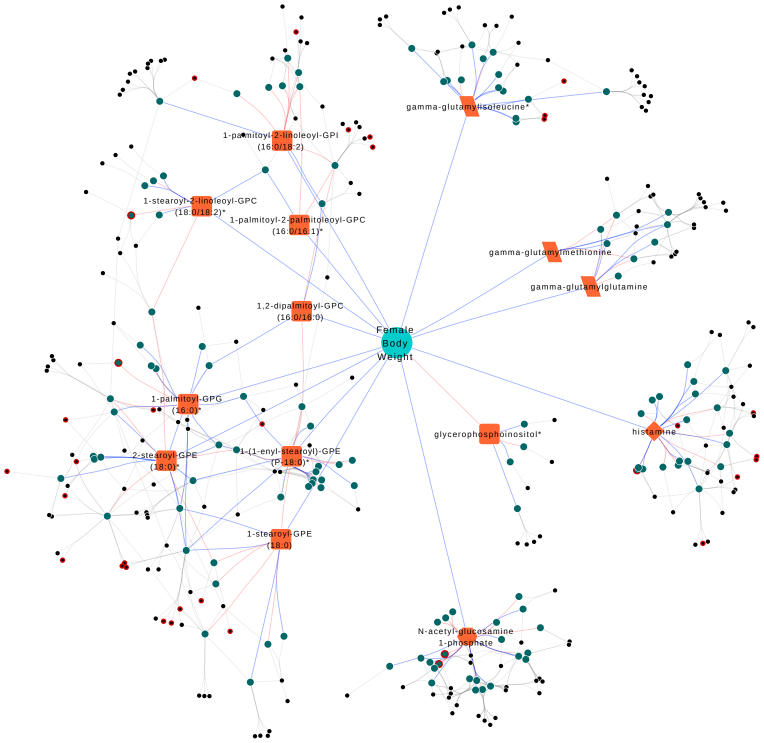
1-palmitoyl-2-linoleoyl-GPI
(16:0/18:2)
gamma-glutamylisoleucine*
1-stearoyl-2-linoleoyl-GPC
(18:0/18:2)*
1-palmitoyl-2-palmitoleoyl-GPC
(16:0/16:1)*
gamma-glutamylmethionine
gamma-glutamylglutamine
1,2-dipalmitoyl-GPC
(16:0/16:0)
Female
Body
Weight
1-palmitoyl-GPG
(16:0)*
glycerophosphoinositol*
histamine
2-stearoyl-GPE
(18:0)*
1-(1-enyl-stearoyl)-GPE
(P-18:0)*
1-stearoyl-GPE
(18:0)
N-acetyl-glucosamine
1-phosphate

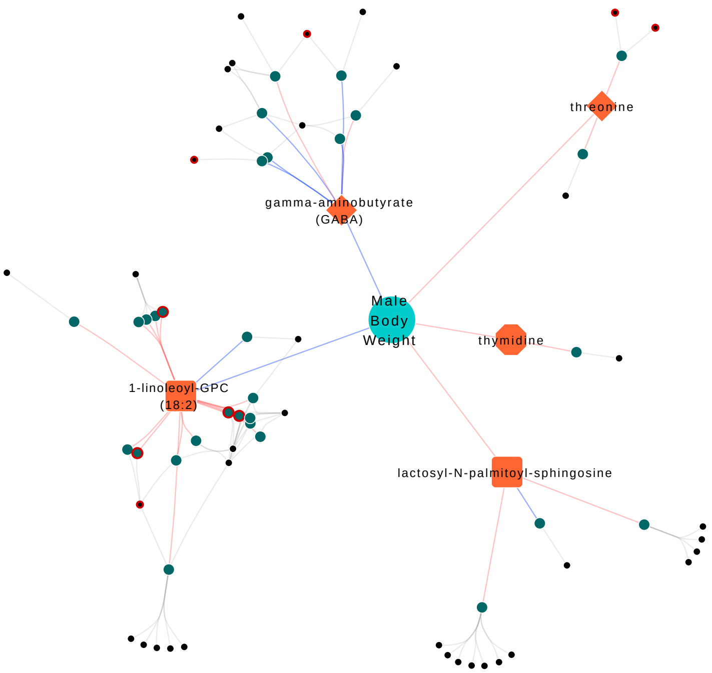
gamma-aminobutyrate
(GABA)
threonine
Male
Body
Weight
thymidine
1-linoleoyl-GPC
(18:2)
lactosyl-N-palmitoyl-sphingosine

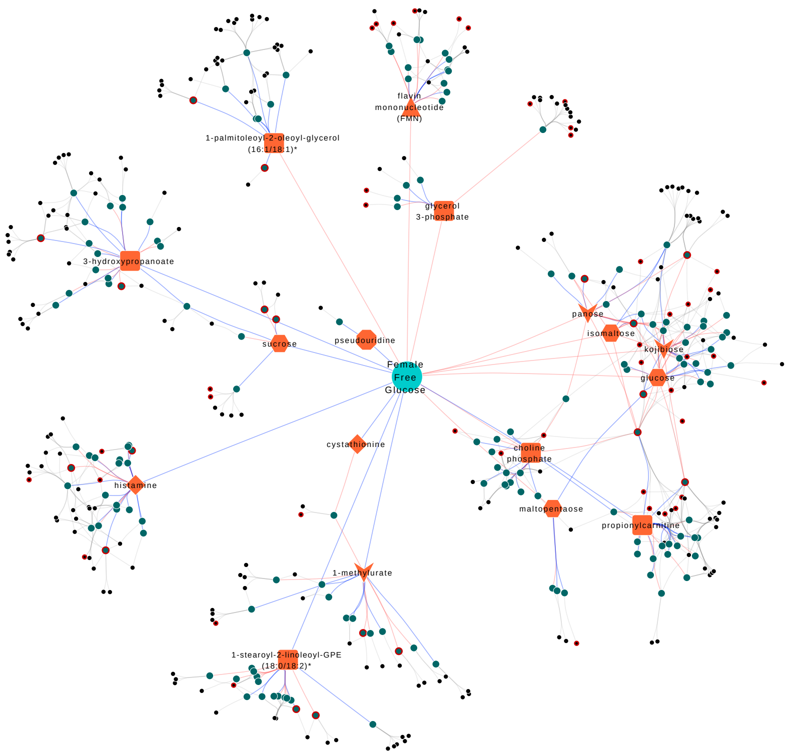
1-palmitoleoyl-2-oleoyl-glycerol (16:1/18:1)*
flavin mononucleotide (FMN)
glycerol 3-phosphate
3-hydroxypropanoate
pseudouridine
sucrose
Female Free Glucose
panose
isomaltose
kojibiose
glucose
cystathionine
histamine
choline phosphate
maltopentaose
propionylcarnitine
1-methylurate
1-stearoyl-2-linoleoyl-GPE (18:0/18:2)*

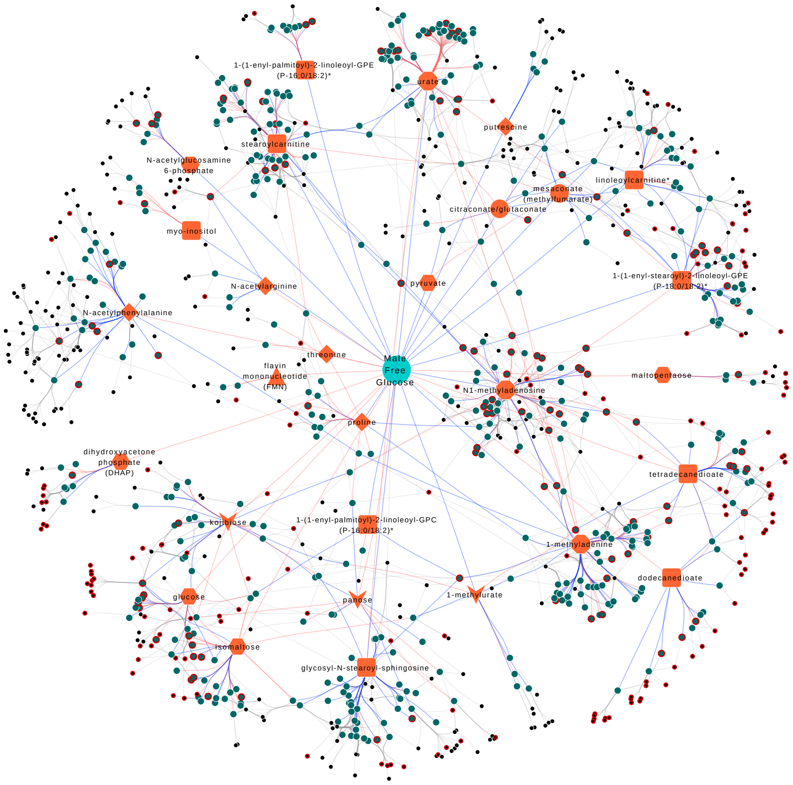

1-(1-enyl-palmitoyl)-2-linoleoyl-GPE
(P-16:0/18:2)*
urate
stearoylcarnitine
putrescine
N-acetylglucosamine
6-phosphate
mesaconate
(methylfumarate)
linoleoylcarnitine*
citraconate/glutaconate
myo-inositol
1-(1-enyl-stearoyl)-2-linoleoyl-GPE
(P-18:0/18:2)*
N-acetylarginine
pyruvate
N-acetylphenylalanine
threonine
Male
Free
Glucose
flavin
mononucleotide
(FMN)
maltopentaose
N1-methyladenosine
proline
dihydroxyacetone
phosphate
(DHAP)
tetradecanedioate
kojibiose
1-(1-enyl-palmitoyl)-2-linoleoyl-GPC
(P-16:0/18:2)*
1-methyladenine
dodecanedioate
glucose
panose
1-methylurate
isomaltose
glycosyl-N-stearoyl-sphingosine

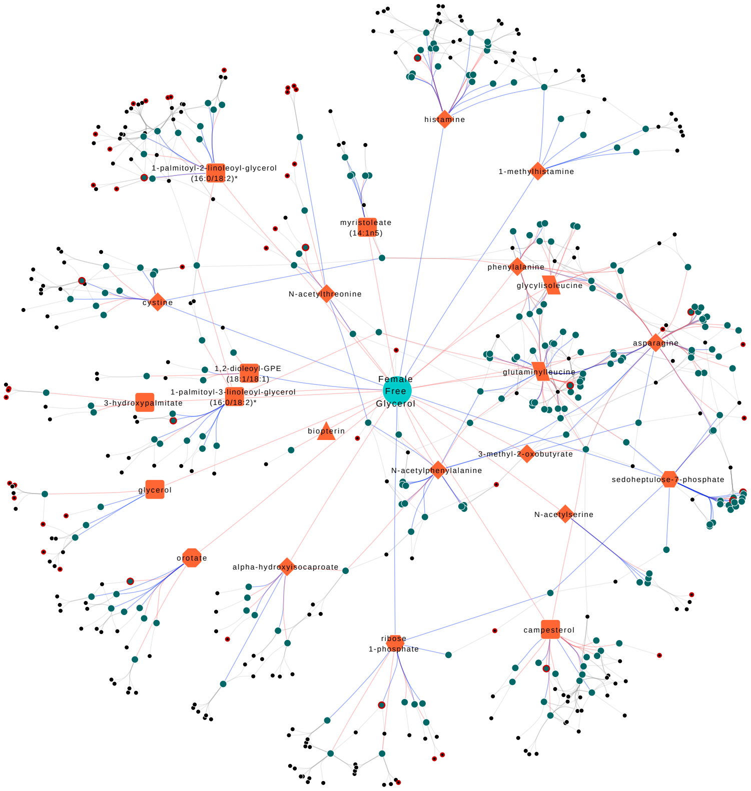

histamine
1-methylhistamine
1-palmitoyl-2-linoleoyl-glycerol
(16:0/18:2)*
myristoleate
(14:1n5)
phenylalanine
glycylisoleucine
cystine
N-acetylthreonine
asparagine
1,2-dioleoyl-GPE
(18:1/18:1)
glutaminylleucine
Female
Free
Glycerol
3-hydroxypalmitate
1-palmitoyl-3-linoleoyl-glycerol
(16:0/18:2)*
biopterin
3-methyl-2-oxobutyrate
N-acetylphenylalanine
sedoheptulose-7-phosphate
glycerol
N-acetylserine
orotate
alpha-hydroxyisocaproate
campesterol
ribose
1-phosphate

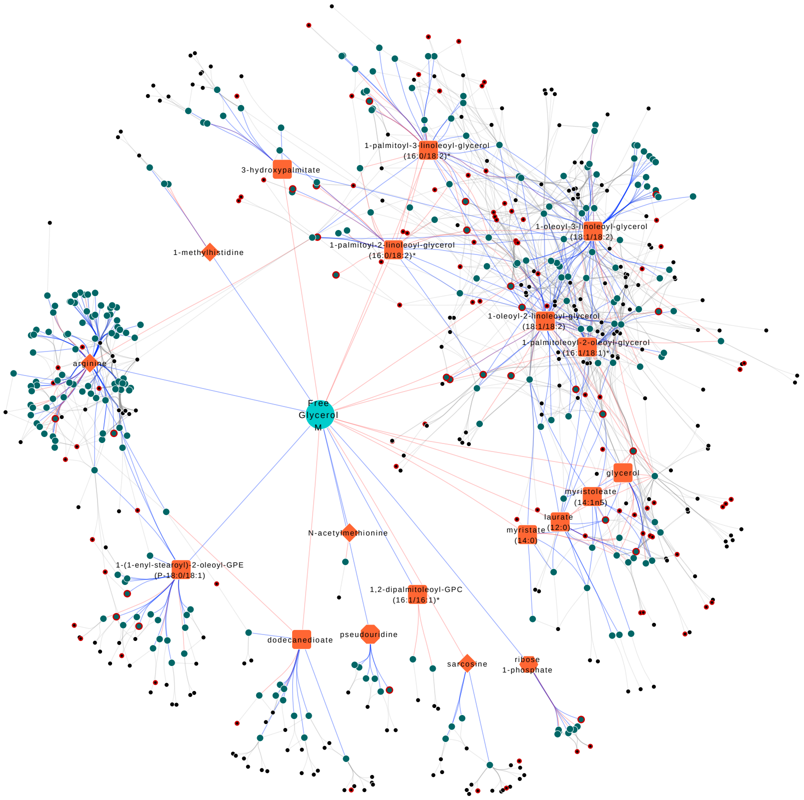
1-palmitoyl-3-linoleoyl-glycerol
(16:0/18:2)*
3-hydroxypalmitate
1-oleoyl-3-linoleoyl-glycerol
(18:1/18:2)
1-palmitoyl-2-linoleoyl-glycerol
(16:0/18:2)*
1-methylhistidine
1-oleoyl-2-linoleoyl-glycerol
(18:1/18:2)
1-palmitoleoyl-2-oleoyl-glycerol
(16:1/18:1)*
arginine
Free
Glycerol
M
glycerol
myristoleate
(14:1n5)
laurate
(12:0)
myristate
(14:0)
N-acetylmethionine
1-(1-enyl-stearoyl)-2-oleoyl-GPE
(P-18:0/18:1)
1,2-dipalmitoleoyl-GPC
(16:1/16:1)*
dodecanedioate
pseudouridine
sarcosine
ribose
1-phosphate

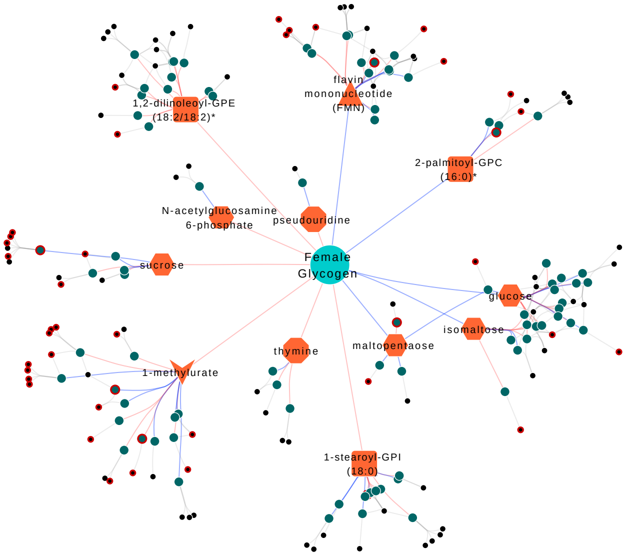

Female Glycogen
1,2-dilinoleoyl-GPE (18:2/18:2)*
flavin mononucleotide (FMN)
2-palmitoyl-GPC (16:0)*
N-acetylglucosamine 6-phosphate
pseudouridine
sucrose
glucose
isomaltose
maltopentaose
thymine
1-methylurate
1-stearoyl-GPI (18:0)

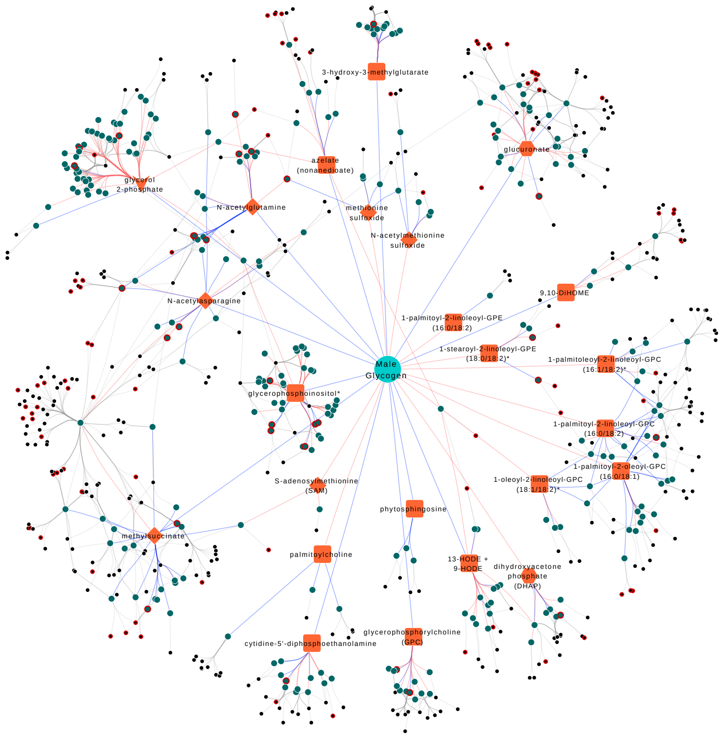

3-hydroxy-3-methylglutarate
azelate (nonanedioate)
glycerol 2-phosphate
N-acetylglutamine
methionine sulfoxide
N-acetylmethionine sulfoxide
glucuronate
9,10-DiHOME
N-acetylasparagine
1-palmitoyl-2-linoleoyl-GPE (16:0/18:2)
1-stearoyl-2-linoleoyl-GPE (18:0/18:2)*
1-palmitoleoyl-2-linoleoyl-GPC (16:1/18:2)*
Male Glycogen
glycerophosphoinositol*
1-palmitoyl-2-linoleoyl-GPC (16:0/18:2)
1-palmitoyl-2-oleoyl-GPC (16:0/18:1)
1-oleoyl-2-linoleoyl-GPC (18:1/18:2)*
S-adenosylmethionine (SAM)
phytosphingosine
methylsuccinate
palmitoylcholine
13-HODE + 9-HODE
dihydroxyacetone phosphate (DHAP)
glycerophosphorylcholine (GPC)
cytidine-5'-diphosphoethanolamine

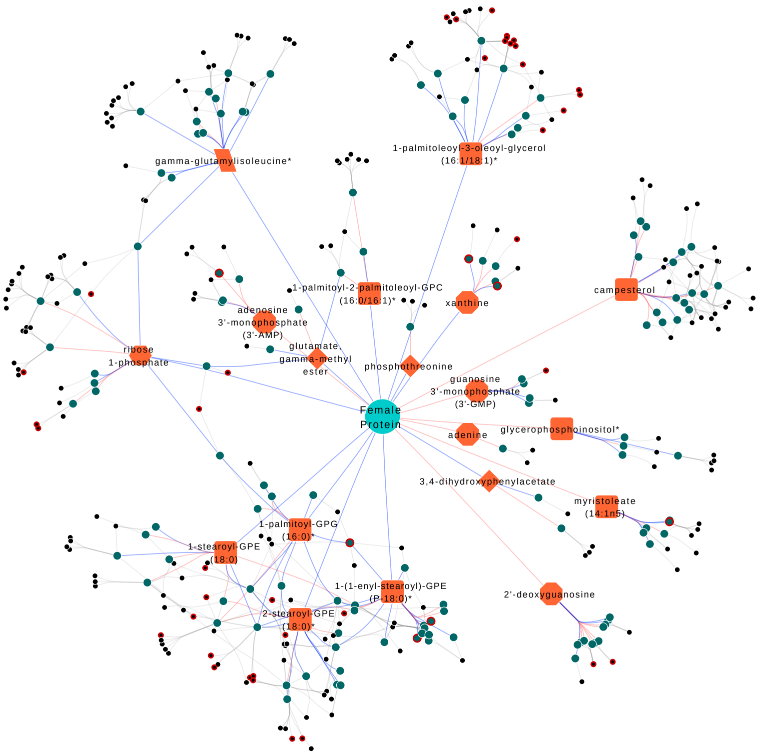

gamma-glutamylisoleucine*
1-palmitoleoyl-3-oleoyl-glycerol
(16:1/18:1)*
1-palmitoyl-2-palmitoleoyl-GPC
(16:0/16:1)*
xanthine
campesterol
adenosine
3'-monophosphate
(3'-AMP)
ribose
1-phosphate
glutamate,
gamma-methyl
ester
phosphothreonine
guanosine
3'-monophosphate
(3'-GMP)
Female
Protein
adenine
glycerophosphoinositol*
3,4-dihydroxyphenylacetate
myristoleate
(14:1n5)
1-palmitoyl-GPG
(16:0)*
1-stearoyl-GPE
(18:0)
1-(1-enyl-stearoyl)-GPE
(P-18:0)*
2'-deoxyguanosine
2-stearoyl-GPE
(18:0)*

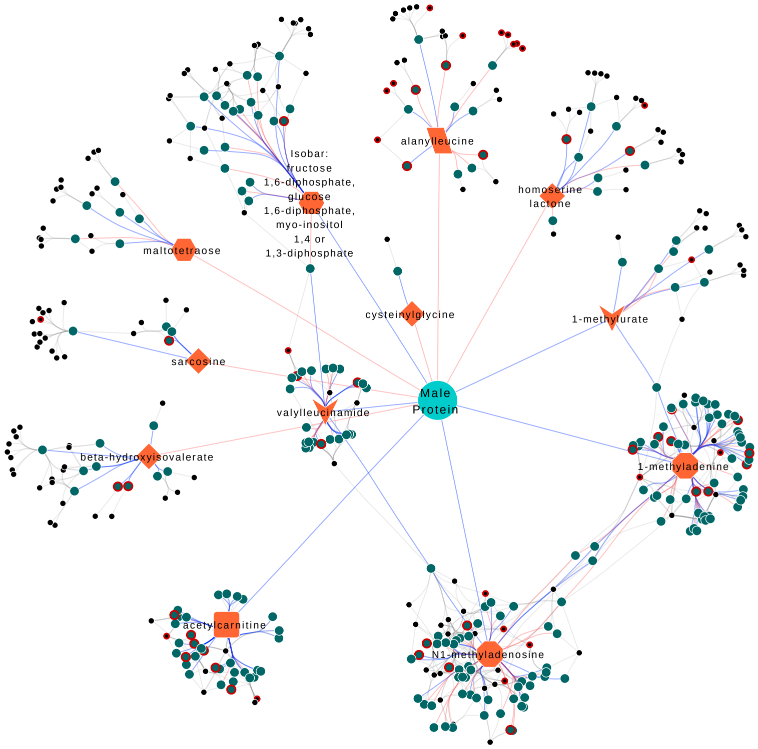
Isobar: fructose 1,6-diphosphate, glucose 1,6-diphosphate, myo-inositol 1,4 or 1,3-diphosphate
alanylleucine
homoserine lactone
maltotetraose
cysteinylglycine
1-methylurate
sarcosine
Male Protein
valylleucinamide
beta-hydroxyisovalerate
1-methyladenine
acetylcarnitine
N1-methyladenosine

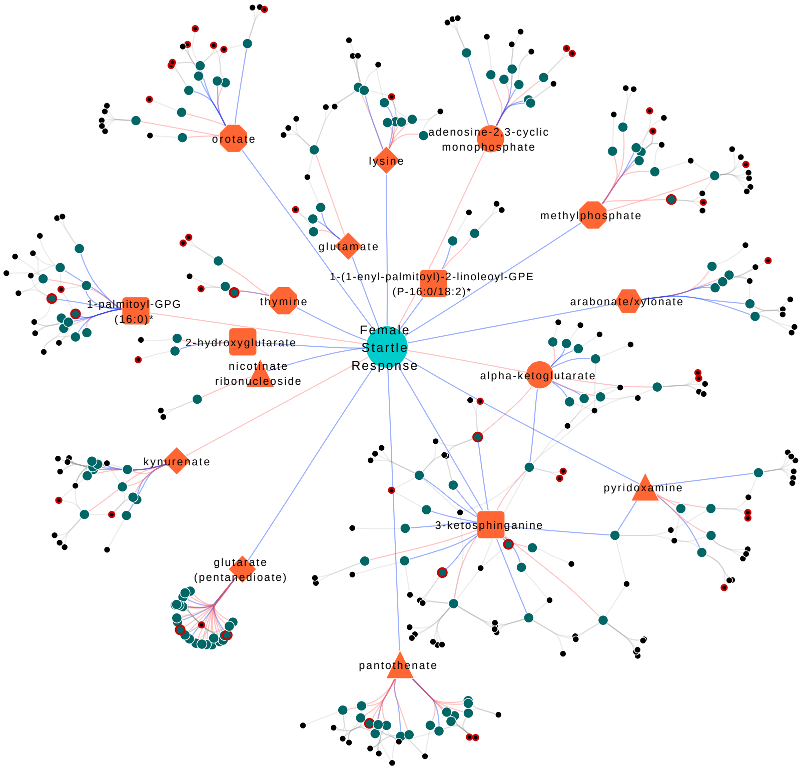
orotate
lysine
adenosine-2,3-cyclic monophosphate
methylphosphate
glutamate
1-(1-enyl-palmitoyl)-2-linoleoyl-GPE (P-16:0/18:2)*
thymine
1-palmitoyl-GPG (16:0)*
arabonate/xylonate
2-hydroxyglutarate
Female Startle Response
nicotinate ribonucleoside
alpha-ketoglutarate
kynurenate
pyridoxamine
3-ketosphinganine
glutarate (pentanedioate)
pantothenate

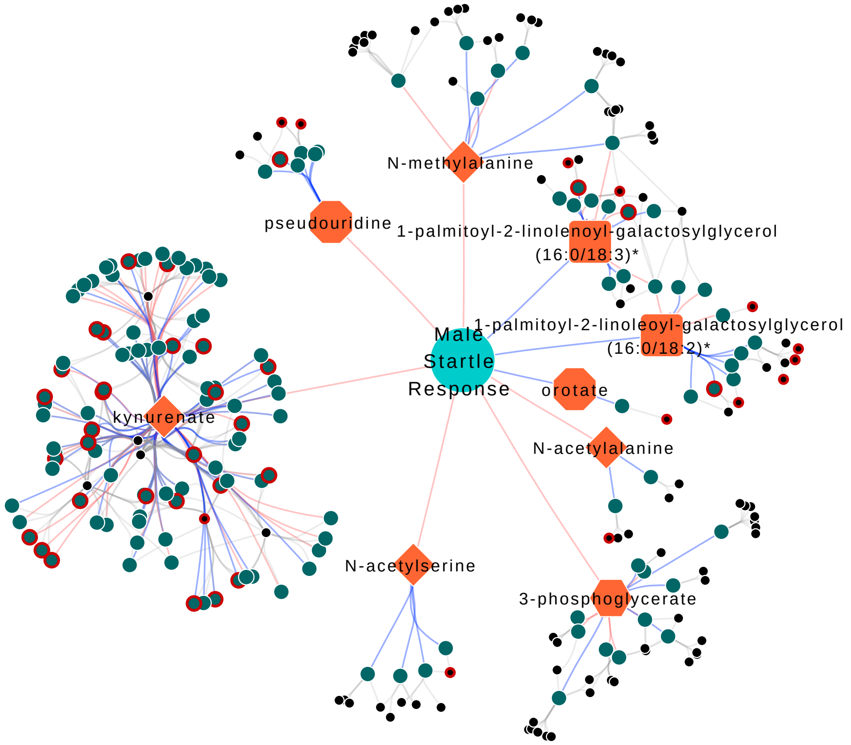

N-methylalanine
pseudouridine
1-palmitoyl-2-linolenoyl-galactosylglycerol (16:0/18:3)*
1-palmitoyl-2-linoleoyl-galactosylglycerol (16:0/18:2)*
Male Startle Response
orotate
kynurenate
N-acetylalanine
N-acetylserine
3-phosphoglycerate

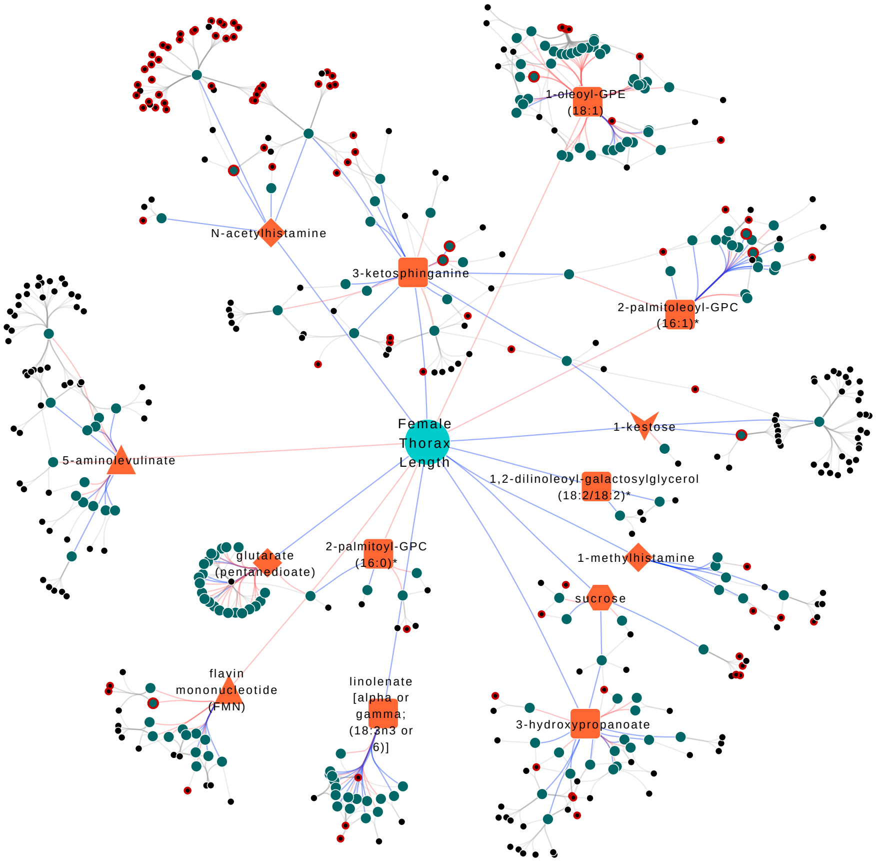

Female Thorax Length
1-oleoyl-GPE (18:1)
N-acetylhistamine
3-ketosphinganine
2-palmitoleoyl-GPC (16:1)*
1-kestose
5-aminolevulinate
1,2-dilinoleoyl-galactosylglycerol (18:2/18:2)*
glutarate (pentanedioate)
2-palmitoyl-GPC (16:0)*
1-methylhistamine
sucrose
flavin mononucleotide (FMN)
linolenate [alpha or gamma; (18:3n3 or 6)]
3-hydroxypropanoate

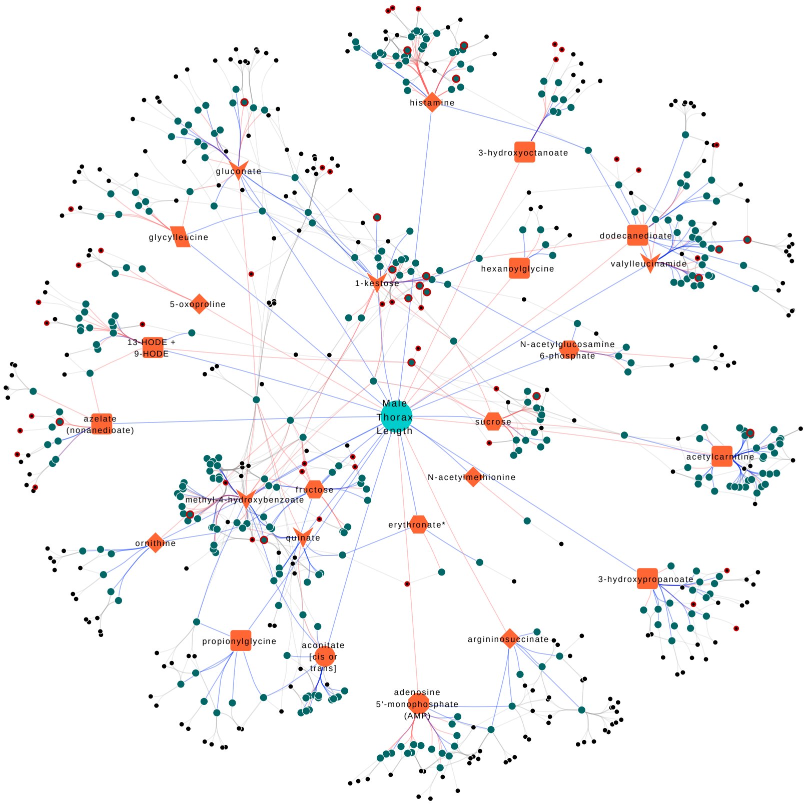
histamine
3-hydroxyoctanoate
gluconate
glycylleucine
dodecanedioate
valylleucinamide
hexanoylglycine
1-kestose
5-oxoproline
13-HODE +
9-HODE
N-acetylglucosamine
6-phosphate
Male
Thorax
Length
azelate
(nonanedioate)
sucrose
acetylcarnitine
N-acetylmethionine
methyl-4-hydroxybenzoate
fructose
erythronate*
ornithine
quinate
3-hydroxypropanoate
propionylglycine
aconitate
[cis or
trans]
argininosuccinate
adenosine
5'-monophosphate
(AMP)

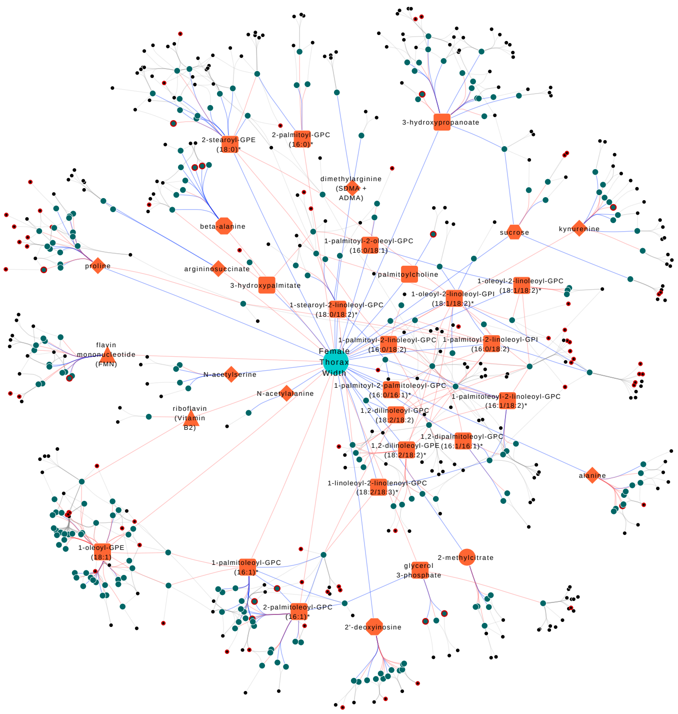

Female Thorax Width
3-hydroxypropanoate
2-stearoyl-GPE (18:0)*
2-palmitoyl-GPC (16:0)*
dimethylarginine (SDMA + ADMA)
beta-alanine
sucrose
kynurenine
1-palmitoyl-2-oleoyl-GPC (16:0/18:1)
proline
argininosuccinate
palmitoylcholine
3-hydroxypalmitate
1-oleoyl-2-linoleoyl-GPI (18:1/18:2)*
1-oleoyl-2-linoleoyl-GPC (18:1/18:2)*
1-stearoyl-2-linoleoyl-GPC (18:0/18:2)*
1-palmitoyl-2-linoleoyl-GPC (18:0/18:2)
1-palmitoyl-2-linoleoyl-GPI (16:0/18:2)
flavin mononucleotide (FMN)
N-acetylserine
1-palmitoyl-2-palmitoleoyl-GPC (16:0/16:1)*
N-acetylalanine
1-palmitoleoyl-2-linoleoyl-GPC (16:1/18:2)*
riboflavin (Vitamin B2)
1,2-dilinoleoyl-GPC (18:2/18:2)
1,2-dipalmitoleoyl-GPC (16:1/16:1)*
1,2-dilinoleoyl-GPE (18:2/18:2)*
alanine
1-linoleoyl-2-linolenoyl-GPC (18:2/18:3)*
1-oleoyl-GPE (18:1)
2-methylcitrate
1-palmitoleoyl-GPC (16:1)*
glycerol 3-phosphate
2-palmitoleoyl-GPC (16:1)*
2'-deoxyinosine

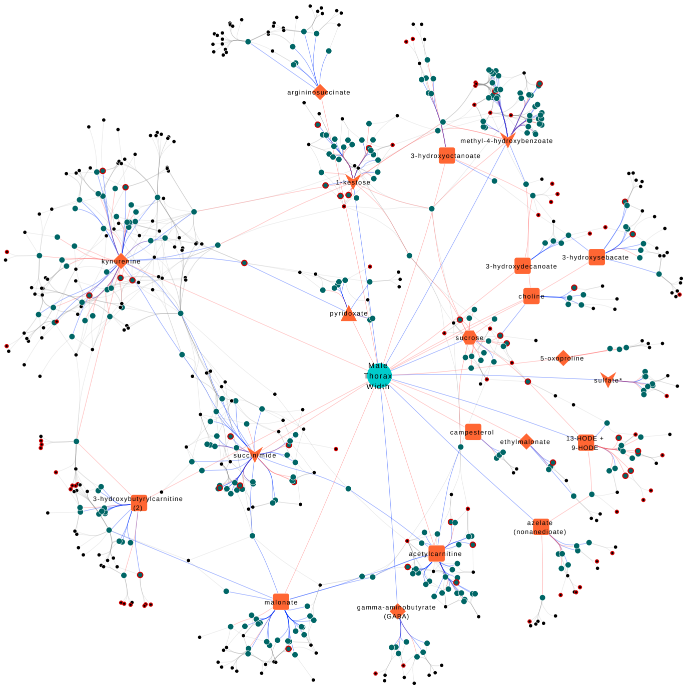
argininosuccinate
3-hydroxyoctanoate
methyl-4-hydroxybenzoate
1-kestose
kynurenine
3-hydroxydecanoate
3-hydroxysebacate
choline
pyridoxate
sucrose
5-oxoproline
Male Thorax Width
sulfate*
campesterol
ethylmalonate
13-HODE + 9-HODE
succinimide
3-hydroxybutyrylcarnitine (2)
azelate (nonanedioate)
acetylcarnitine
malonate
gamma-aminobutyrate (GABA)

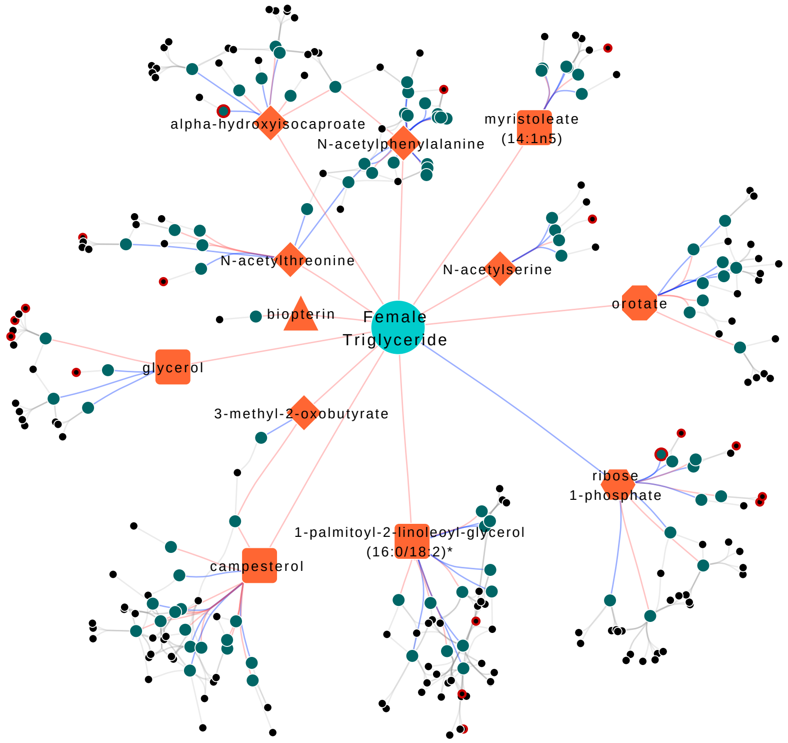

alpha-hydroxyisocaproate
N-acetylphenylalanine
myristoleate (14:1n5)
N-acetylthreonine
N-acetylserine
orotate
biopterin
Female Triglyceride
glycerol
3-methyl-2-oxobutyrate
ribose 1-phosphate
1-palmitoyl-2-linoleoyl-glycerol (16:0/18:2)*
campesterol

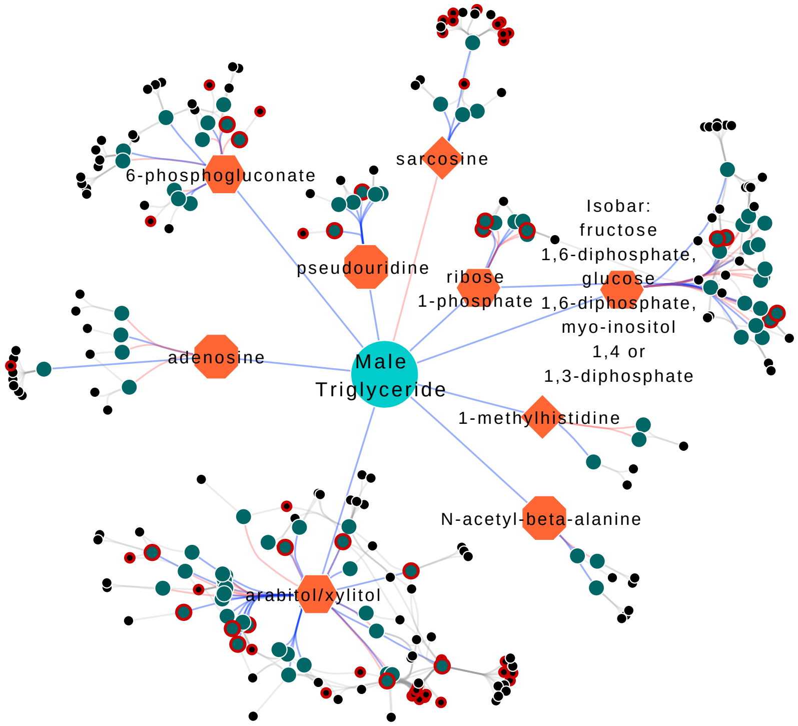
Male
Triglyceride
6-phosphogluconate
sarcosine
pseudouridine
ribose
1-phosphate
Isobar:
fructose
1,6-diphosphate,
glucose
1,6-diphosphate,
myo-inositol
1,4 or
1,3-diphosphate
adenosine
1-methylhistidine
N-acetyl-beta-alanine
arabitol/xylitol

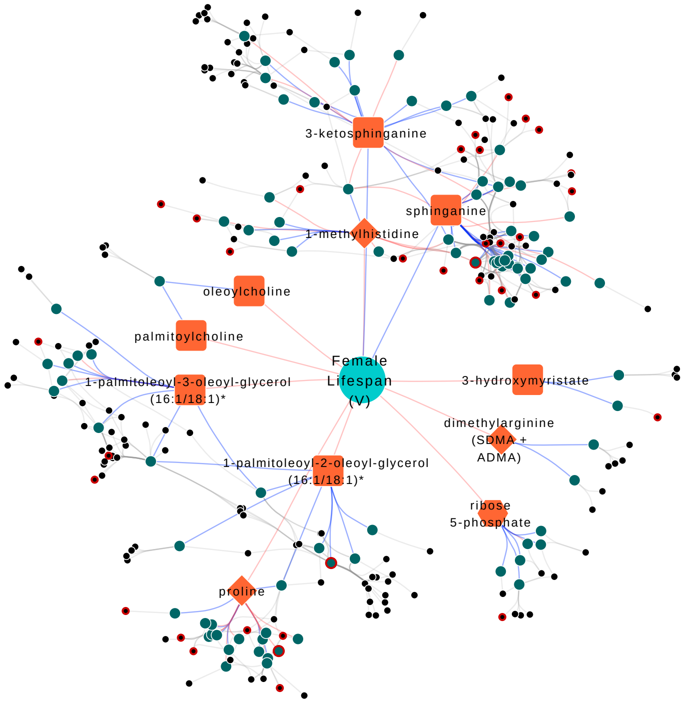
3-ketosphinganine
sphinganine
1-methylhistidine
oleoylcholine
palmitoylcholine
Female Lifespan (V)
1-palmitoleoyl-3-oleoyl-glycerol (16:1/18:1)*
3-hydroxymyristate
dimethylarginine (SDMA + ADMA)
1-palmitoleoyl-2-oleoyl-glycerol (16:1/18:1)*
ribose 5-phosphate
proline

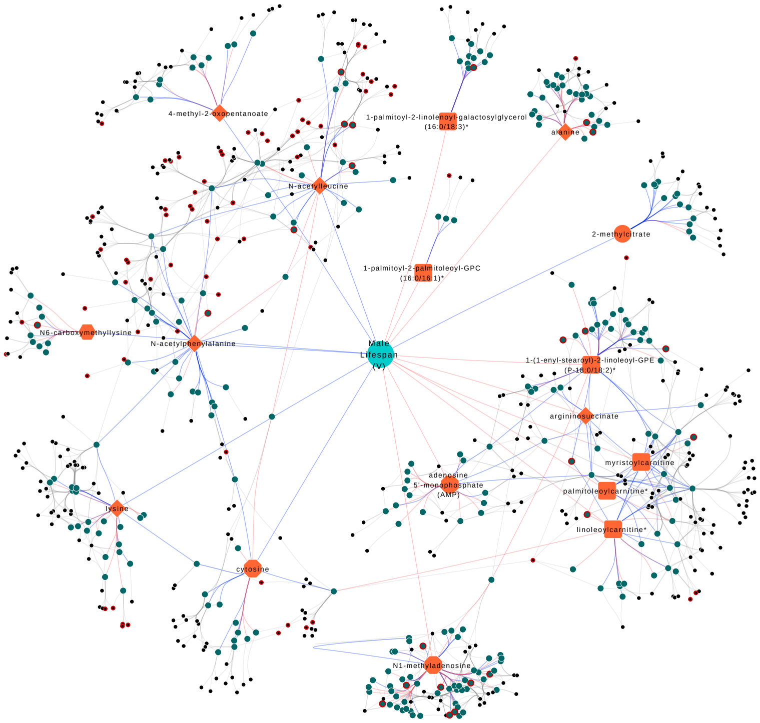
4-methyl-2-oxopentanoate
1-palmitoyl-2-linolenoyl-galactosylglycerol
(16:0/18:3)*
alanine
N-acetylleucine
2-methylcitrate
1-palmitoyl-2-palmitoleoyl-GPC
(16:0/16:1)*
N6-carboxymethyllysine
N-acetylphenylalanine
Male
Lifespan
(V)
1-(1-enyl-stearoyl)-2-linoleoyl-GPE
(P-18:0/18:2)*
argininosuccinate
myristoylcarnitine
adenosine
5'-monophosphate
(AMP)
palmitoleoylcarnitine*
linoleoylcarnitine*
lysine
cytosine
N1-methyladenosine

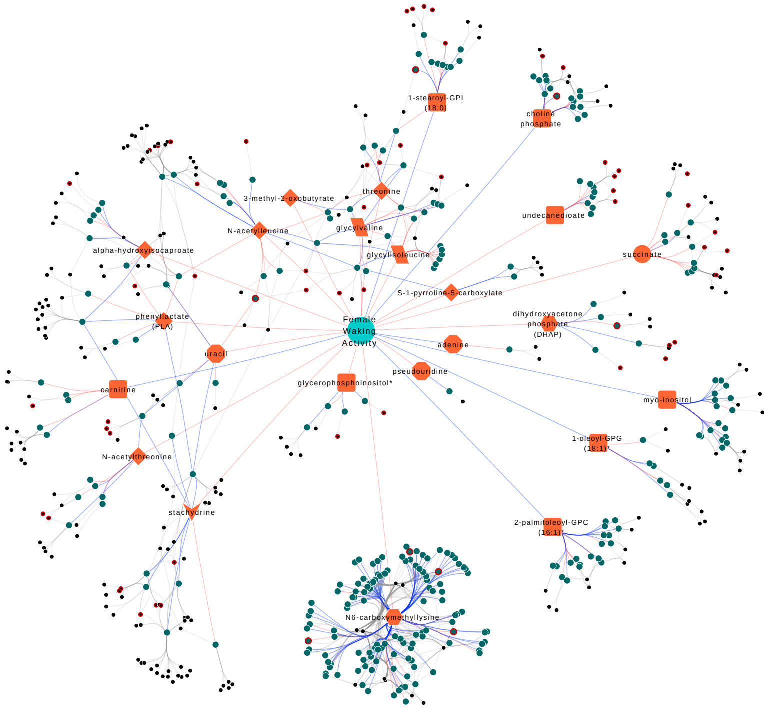
1-stearoyl-GPI
(18:0)
choline
phosphate
3-methyl-2-oxobutyrate
threonine
undecanedioate
N-acetylleucine
glycylvaline
alpha-hydroxyisocaproate
glycylisoleucine
succinate
S-1-pyrroline-5-carboxylate
phenyllactate
(PLA)
Female
Waking
Activity
dihydroxyacetone
phosphate
(DHAP)
uracil
adenine
pseudouridine
glycerophosphoinositol*
carnitine
myo-inositol
1-oleoyl-GPG
(18:1)*
N-acetylthreonine
stachydrine
2-palmitoleoyl-GPC
(16:1)*
N6-carboxymethyllysine

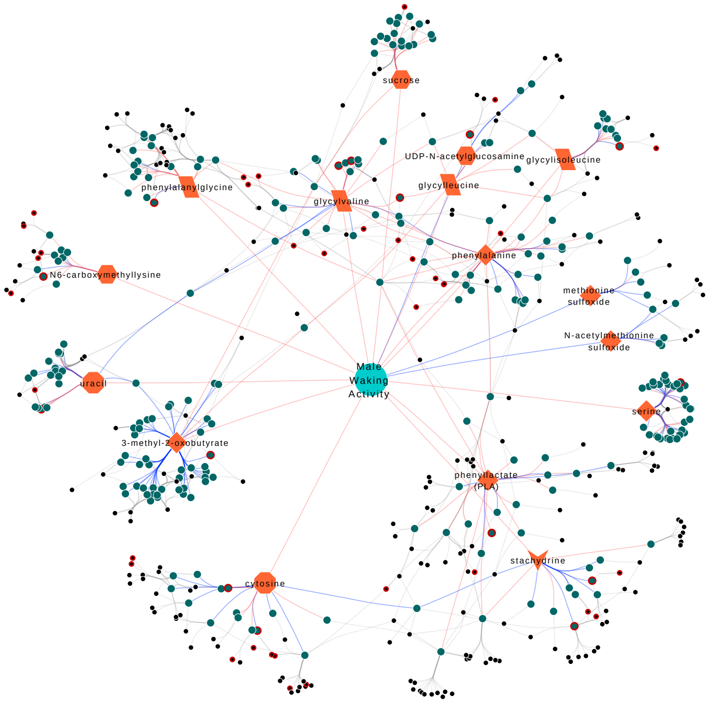

sucrose
UDP-N-acetylglucosamine
glycylisoleucine
phenylalanylglycine
glycylvaline
glycylleucine
phenylalanine
N6-carboxymethyllysine
methionine sulfoxide
N-acetylmethionine sulfoxide
Male Waking Activity
uracil
serine
3-methyl-2-oxobutyrate
phenyllactate (PLA)
stachydrine
cytosine

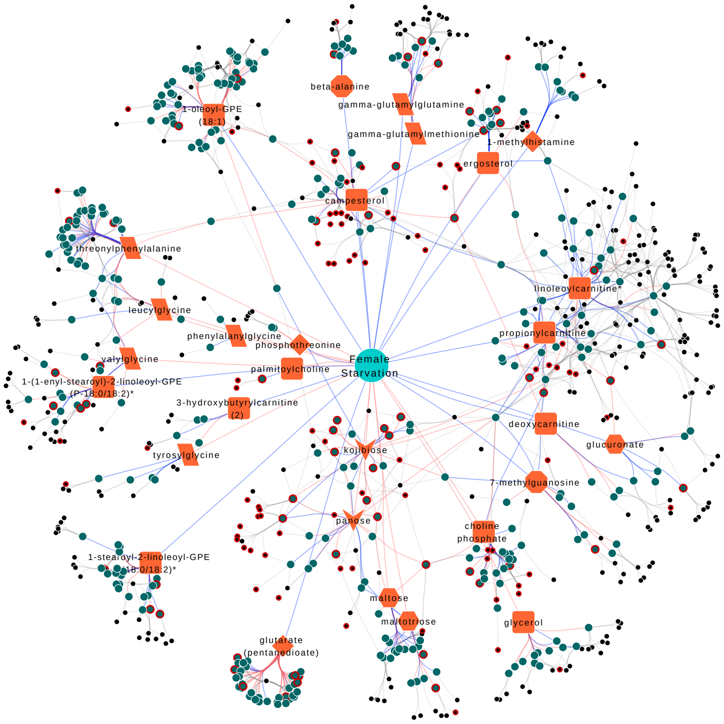
beta-alanine
gamma-glutamylglutamine
gamma-glutamylmethionine
1-methylhistamine
1-oleoyl-GPE
(18:1)
ergosterol
campesterol
threonylphenylalanine
linoleoylcarnitine*
leucylglycine
propionylcarnitine
phenylalanylglycine
phosphothreonine
Female
Starvation
valylglycine
palmitoylcholine
1-(1-enyl-stearoyl)-2-linoleoyl-GPE
(P-18:0/18:2)*
3-hydroxybutyrylcarnitine
(2)
deoxycarnitine
glucuronate
kojibiose
tyrosylglycine
7-methylguanosine
panose
choline
phosphate
1-stearoyl-2-linoleoyl-GPE
(18:0/18:2)*
maltose
maltotriose
glycerol
glutarate
(pentanedioate)

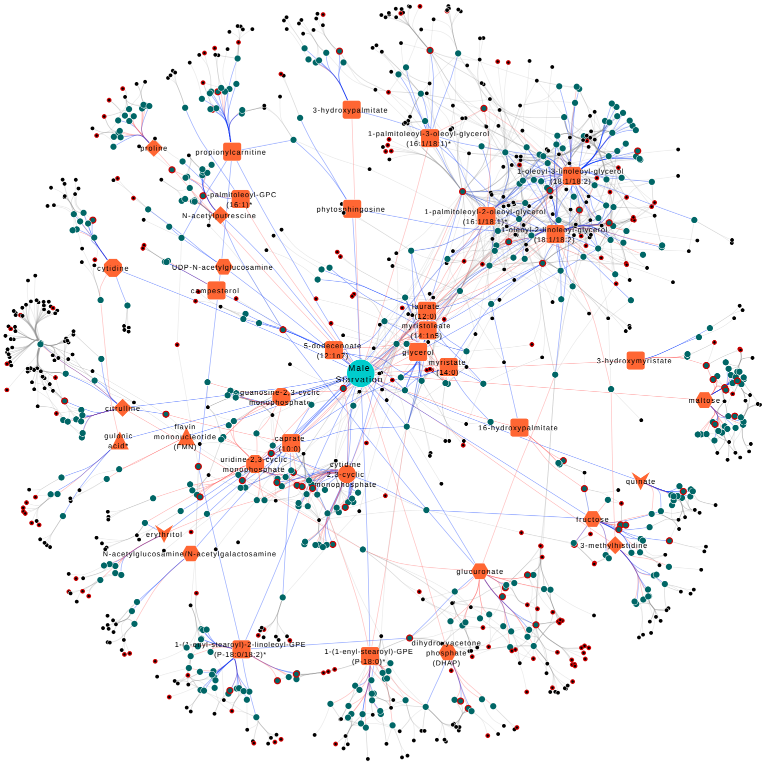
3-hydroxypalmitate
1-palmitoleoyl-3-oleoyl-glycerol
(16:1/18:1)*
proline
propionylcarnitine
1-oleoyl-3-linoleoyl-glycerol
(18:1/18:2)
palmitoleoyl-GPC
(16:1)*
N-acetylputrescine
phytosphingosine
1-palmitoleoyl-2-oleoyl-glycerol
(16:1/18:1)
1-oleoyl-2-linoleoyl-glycerol
(18:1/18:2)
cytidine
UDP-N-acetylglucosamine
campesterol
laurate
(12:0)
myristoleate
(14:1n5)
5-dodecenoate
(12:1n7)
glycerol
myristate
(14:0)
3-hydroxymyristate
Male
Starvation
guanosine-2,3-cyclic
monophosphate
maltose
citrulline
flavin
mononucleotide
(FMN)
16-hydroxypalmitate
gulonic
acid
caprate
(10:0)
uridine-2,3-cyclic
monophosphate
cytidine
2,3-cyclic
monophosphate
quinate
fructose
erythritol
3-methylhistidine
N-acetylglucosamine/N-acetylgalactosamine
glucuronate
1-(1-enyl-stearoyl)-2-linoleoyl-GPE
(P-18:0/18:2)*
1-(1-enyl-stearoyl)-GPE
(P-18:0)
dihydroxyacetone
phosphate
(DHAP)

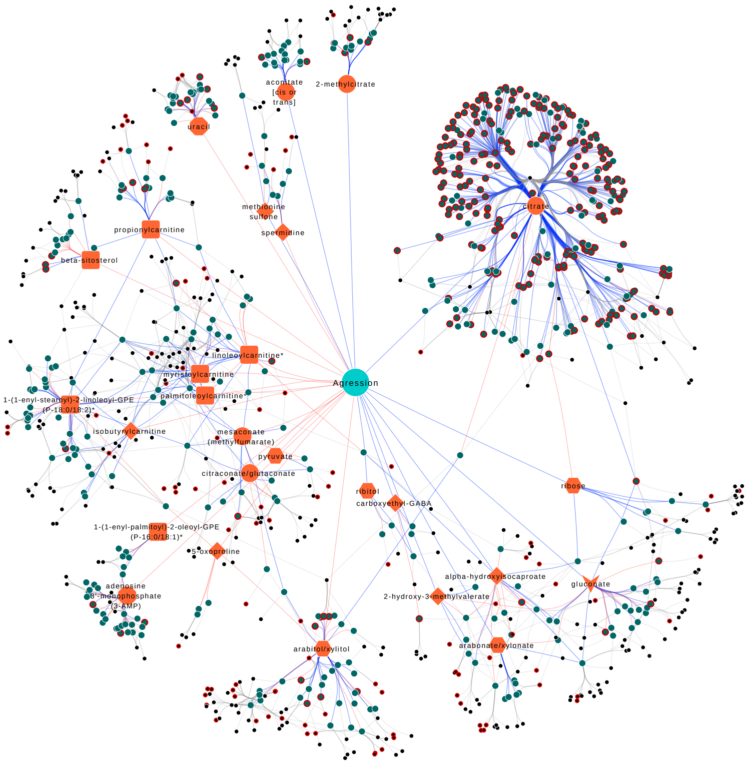
2-methylcitrate
aconitate
[cis or
trans]
uracil
methionine
sulfone
spermidine
propionylcarnitine
beta-sitosterol
citrate
linoleoylcarnitine*
myristoylcarnitine
palmitoleoylcarnitine*
Agression
1-(1-enyl-stearoyl)-2-linoleoyl-GPE
(P-18:0/18:2)*
isobutyrylcarnitine
mesaconate
(methylfumarate)
pyruvate
citraconate/glutaconate
ribitol
carboxyethyl-GABA
ribose
1-(1-enyl-palmitoyl)-2-oleoyl-GPE
(P-16:0/18:1)*
5-oxoproline
adenosine
3'-monophosphate
(3-AMP)
alpha-hydroxyisocaproate
gluconate
2-hydroxy-3-methylvalerate
arabonate/xylonate
arabitol/xylitol
